# High School Follow-Up of the Dating Matters® RCT: Effects on Teen Dating Violence and Relationship Behaviors

**DOI:** 10.1007/s11121-024-01648-z

**Published:** 2024-03-08

**Authors:** Phyllis Holditch Niolon, Lianne F. Estefan, Sarah DeGue, Vi D. Le, Allison J. Tracy, Colleen Ray, Daniel Bontempo, Todd D. Little, Alana M. Vivolo-Kantor, Natasha Latzman, Bruce Taylor, Andra Tharp

**Affiliations:** 1 Division of Violence Prevention, National Center for Injury Prevention and Control, Centers for Disease Control and Prevention, 4770 Buford Highway NE, Atlanta, GA30341 S106-10 USA; 2 TJFACT Inc, Contractor for the Division of Violence Prevention, Atlanta, GA USA; 3 College of Education, Texas Tech University, Lubbock, TX USA; 4 North-West University, Vanderbijlpark, South Africa; 5 Division of Overdose Prevention, National Center for Injury Prevention and Control, Centers for Disease Control and Prevention, Atlanta, GA USA; 6 Harvard, Massachusetts, MA USA; 7 NORC, University of Chicago, Chicago, IL USA; 8 Sexual Assault Prevention and Research Office, Department of Defense, Washington, DC USA

**Keywords:** Teen dating violence, Intervention research, Relationship skills

## Abstract

Teen dating violence (TDV) is a significant public health problem that can have lifelong consequences. Using a longitudinal, cluster randomized controlled trial (RCT), this study examines whether the *Dating Matters* comprehensive prevention model, implemented in middle school, prevented TDV and negative relationship behaviors and promoted positive relationship behaviors in high school (9th–11th grades), when compared with a standard of care intervention. *Dating Matters* includes programs for sixth to eighth grade youth and their parents, training for school staff, a youth communications program, and policy and data activities implemented in the community. Self-report survey data were collected from students in 46 middle schools that were randomly assigned to condition within site. Students completed two surveys (fall and spring) in each middle school grade and a single survey in the spring of each high school grade. This study examined self-reported TDV perpetration and victimization, use of negative conflict resolution strategies, and positive relationship skills in the high school follow-up. While varying patterns emerged, latent panel models demonstrated significant program effects for all outcomes. *Dating Matters* students reported 19% reduced risk for TDV perpetration, 24% reduced risk for TDV victimization, 7% reduced risk for use of negative conflict strategies, and 3% more use of positive relationship skills, on average across time and cohort, than standard of care students. On average, *Dating Matters*, implemented in middle school, continued to be more effective at reducing TDV perpetration, TDV victimization, and use of negative conflict resolution strategies in high school than an evidence-based comparison program.

Trial Registration: clinicaltrials.gov Identifier: NCT01672541.

**Supplementary Information:**

The online version contains supplementary material available at 10.1007/s11121-024-01648-z.

The prevention of teen dating violence (TDV) and related risk behaviors is a critical public health endeavor, not only because of the immediate consequences of TDV for adolescents but also due to an increased risk of experiencing intimate partner violence (IPV) across the lifespan (Capaldi et al., 2012; Exner-Cortens et al., 2017). TDV and IPV include physical, sexual, emotional/psychological violence, and stalking by a current or former dating/intimate partner (Breiding et al., 2015) National estimates from 2019 indicate that roughly 8% of US high school students who dated in the past year experienced physical dating violence victimization, and about the same proportion of students experienced sexual dating violence (Basile et al., 2020); prevalence estimates from other samples are often even higher (Wincentak et al., 2017). TDV is associated with a host of deleterious consequences for adolescents, including injury, academic problems, substance use, depression, and suicidal ideation (Exner-Cortens et al., 2013, 2017; Foshee et al., 2004a, b; Offenhauer & Buchalter, 2011). TDV also increases risk for IPV in adulthood (Capaldi et al., 2012; Exner-Cortens et al., 2017), which is associated with its own set of negative lifelong consequences. Interventions to prevent TDV should strive not only to prevent TDV in the short term but to ensure the preventive effects persist over time.

Several interventions have demonstrated short-term effectiveness for preventing TDV (De La Rue et al., 2017; Piolanti & Foran, 2021). Yet, to date, only *Fourth R* (Wolfe et al., 2009) and *Safe Dates* (Foshee et al., 2004a, b), both of which are school-based interventions focusing on skills, attitudes, and beliefs, have demonstrated effectiveness for preventing both TDV perpetration and victimization (*Fourth R* only assessed perpetration) beyond a one-year postintervention period. In a follow-up study of Fourth R, adolescents who received the program in 9th grade reported significantly less physical TDV perpetration 2.5 years after the program compared to adolescents in the control group (Wolfe et al., 2009). Adolescents who received *Safe Dates* in 8th grade reported significantly less TDV perpetration and victimization four years after the program compared to adolescents in a control group (Foshee et al., 2004a, b). These longer-term effects are particularly promising because the literature on universal prevention shows that most intervention effects are strongest in the short term and often disappear in long-term follow-ups (Durlak et al., 2011). Decreases in effectiveness of programs as time progresses may be due to insufficient duration of the programs, a focus on strengthening awareness rather than changing social norms and developing behavioral skills, or lack of time to practice such skills so that they become ingrained; therefore, efforts to lengthen programs (or provide booster interventions) and increased efforts to change norms and help youth develop and practice skills may help more interventions demonstrate sustained effects.

## The Current Study

The current study uses high school follow-up data from a randomized controlled trial (RCT) of *Dating Matters: Strategies to Promote Healthy Teen Relationships*® (DM), implemented during middle school, to evaluate its long-term effects on TDV and other relationship behaviors in 9th–11th grade. DM is a comprehensive prevention model that includes unique prevention programs for middle school youth in 6th, 7th, and 8^th^ grade, as well as three unique programs for parents of 6th, 7th, and 8th graders, with each program designed to be developmentally relevant and building on content delivered in earlier grades. In addition, the model includes training for school staff, a youth communications program, and policy and data activities implemented in the community to promote healthy relationships and prevent TDV and related risk behaviors.^1^

DM was evaluated using a longitudinal, comparative effectiveness, multi-site cluster RCT in four US cities. Although some research suggests adolescents living in economically and socially disadvantaged neighborhoods may be at higher risk for TDV (Wincentak et al., 2017), they have been underrepresented in the TDV prevention literature (Teten Tharp et al., 2011). To address this gap, schools included in the Dating Matters RCT were located in urban neighborhoods identified by local health departments as having above average rates of crime and poverty. In middle school, DM was found to prevent TDV perpetration and victimization and reduce the use of negative conflict resolution strategies (ways of resolving a conflict that are ineffective or may lead to higher risk of aggression) (Niolon et al., 2019) and a host of secondary outcomes including other forms of interpersonal aggression and risk behaviors (DeGue et al., 2021; Estefan et al., 2021; Vivolo-Kantor et al., 2021) when compared to an evidence-based, standard-of-care (SC) TDV prevention program implemented in 8th grade only (*Safe Dates*) (Foshee et al., 1998). No effects were found during middle school on positive relationship behaviors (Niolon et al., 2019). Effectiveness during middle school was examined among the two cohorts of students (Cohorts 3 and 4) who had the opportunity for full exposure to DM in 6th, 7th, and 8th grade and, for dating violence outcomes, only among those who reported dating at any point in middle school (Niolon et al., 2019).

The current study follows the same two full-exposure cohorts into high school and examines long-term effects (assessments in grades 9, 10, and 11 after the intervention ended in 8th grade) on the primary outcomes of the RCT, specifically TDV perpetration and victimization, negative conflict resolution strategies, and positive relationship behaviors, among students who reported dating at any point in middle or high school. Because the multiple components of DM focused on helping middle school students develop healthy relationship skills, we hypothesized that the effects of DM would last beyond the intervention and continue to be protective as youth mature and engage in more intimate romantic relationships. Specifically, we hypothesized that students in DM schools, as compared to students in SC schools, would continue to report less TDV perpetration, less TDV victimization, less use of negative conflict resolution strategies, and higher use of positive relationship skills in high school.

## Methods

### Study Design and Analytic Sample

Self-report survey data were collected as part of a nine-wave RCT to evaluate the effectiveness of DM between 2012 and 2018 from students in 46 middle schools. The evaluation contractor randomly assigned schools within each site to either the DM or SC condition using a simple computer-generated random numbers approach so that each school within each site had an equal chance of being assigned to condition^15^ (see online supplement for more details). Students completed two surveys (fall and spring) in each middle school grade (Grades 6, 7, and 8), and a single survey in the spring of each high school grade (Grades 9, 10, and 11). The analytic sample for this study includes students who started 6th grade in either 2012 or 2013 (Cohort 3 and 4), because these students had an opportunity for full exposure to DM in intervention schools during implementation. During the high school follow-up, Cohort 3 was assessed in Grades, 9, 10, and 11. However, due to logistical challenges, Cohort 4 was only assessed in grades 9 and 10. Detailed information on the study design including randomization and full sample are provided elsewhere (Niolon et al., 2016).

**Fig. 1:**
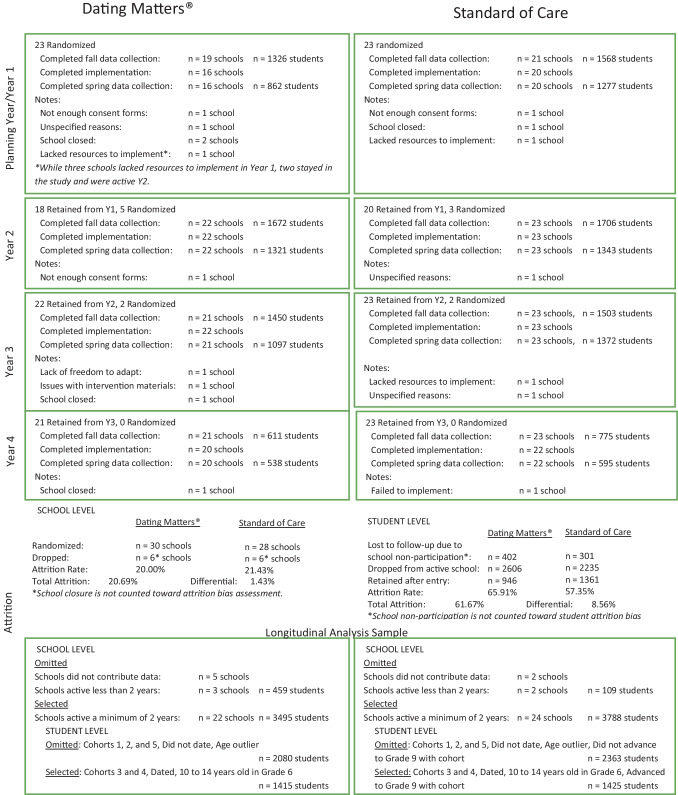
Dating Matters high school follow-up RCT CONSORT diagram

As in the middle school evaluation of the RCT of Dating Matters, we omitted students in schools who did not participate at least two years in either the standard of care (N_schools_ = 2, N_students_ = 58) or *Dating Matters* program (N_schools_ = 3, N_students_ = 240); this decision (discussed in greater detail in Niolon et al., 2019) was based on the fact that schools implementing less than 2 years would have implemented less than half of the 3-year middle school span covered by the DM components and that students from the schools would have less than half of the survey data collection opportunities across the 3 years of middle school; once schools dropped out, we were no longer able to collect data from their students. Schools that were omitted were similar in terms of percentage of students on free/reduced price lunch and racial/ethnic composition but were smaller and had a lower student–teacher ratios than schools that were not omitted. We also omitted students who never reported having dated by Grade 11 (N_SC_ = 202, N_DM_ = 240) because all the outcomes examined in this paper were only measured for students who had dated at some point during middle and high school. A few students who did not advance with their cohort to Grade 9 and students representing age outliers (older than 14 or younger than 10 years of age in the fall of their Grade 6 school year) (N_SC_ = 8, N_DM_ = 8) were also omitted from the analysis sample. Our selection criteria resulted in an analysis sample of 2840 students (*N*_SC_ = 1425; *N*_DM_ = 1415). The analytic sample was balanced with respect to sex (51% female). Most students in the sample identified as Black, non-Hispanic (53%), or Hispanic (29%). The mean age at entry into the study (fall of 6th grade) was 11.9 (*SD* = 0.6). The CONSORT diagram for the analytic sample is in Fig. 1, and sample descriptives by condition, gender, and cohort are provided in eTable 3.

### Measures

#### Teen Dating Violence Perpetration (TDVP) and Victimization (TDVV)

Students who reported dating within the previous four-month period responded to 62 items asking about violence in their primary dating relationship during the past four months. Fifty of these items (25 assessing perpetration and 25 assessing victimization) were from the Conflict in Adolescent Dating Relationships Inventory (Wolfe et al., 2001) The remaining 12 items (6 assessing perpetration and 6 assessing victimization) were from a study evaluating the *Safe Dates* program (Foshee et al., 1998) Together, these items assess five types of TDVP and TDVV using a 4-point scale (1 = never, 2 = seldom, 3 = sometimes, 4 = often): physical abuse (e.g., I threw something at him/her), threatening behaviors (e.g., I deliberately tried to frighten him/her), sexual abuse (e.g., I forced him/her to have sex when he/she didn’t want to), relational abuse (e.g., I said things just to make him/her angry), and emotional abuse (e.g., I insulted him/her with put-downs). Cronbach’s alpha coefficients across time and group ranged from 0.72 to 0.90 for TDVP (*M* = 0.82) and from 0.74 to 0.91 for TDVV (*M* = 0.83).

#### Negative Conflict Resolution Strategies (NCRS)

The use of negative conflict resolution strategies was assessed by the Conflict Resolution Style Inventory (Kurdek, 1994). This study used three subscales, each with four items: Compliance (e.g., not being willing to stick up for myself), Conflict Engagement (e.g., launching personal attacks), and Withdrawal (e.g., remaining silent for long periods of time). These items used a 5-point scale (1 = never, 2 = almost never, 3 = sometimes, 4 = often, 5 = always). Alpha reliabilities ranged across time and group from 0.62 to 0.79 (*M* = 0.71).

#### Positive Relationship Skills (PRS)

Four items from the Supporting Healthy Marriage Study (Miller Gaubert et al., 2012), adapted for this study to reflect pre-teen and teen dating relationships, were used to assess the frequency of the use of positive relationship skills. These items assessed positive interactions with a dating partner (e.g., being honest and working out differences) on a 4-point scale (1 = never, 2 = sometimes, 3 = usually, 4 = always). This outcome is positively worded; a higher score indicates a better outcome. Reliability was marginal for this measure, motivating a latent variable approach; coefficients ranged from 0.59 to 0.74 (*M* = .69).

Our analysis plan centered on the use of a novel modeling approach that is well-suited for dealing with the complexity inherent in this multi-group, multi-wave evaluation design (see the Statistical Analysis section below). To prepare the data for this modeling approach, it is recommended that the outcome scores be adjusted for covariate effects prior to hypothesis testing (Little et al., 2021), using residualized scoring (Little, 2013). Prior to fitting the analysis models, we statistically adjusted the outcome scores with respect to the following covariates (see eMethod for further description of covariate measurement and coding): baseline levels of the outcome variables (Grade 6 fall, prior to program implementation), relative student age difference,^2^ survey administration timing, race/ethnicity, guardianship status, survey assessment mode, site, and reports of having witnessed violence in the home or community. Descriptive statistics and the construction of covariate-adjusted latent variables used in the evaluation models are described in the online supplement (eTables 1 and 2). Descriptive characteristics and equivalency tests of the covariates are in eTable 3.

### Statistical Analysis

Each of the four primary outcomes was evaluated separately. As with the middle school primary outcomes paper (Niolon et al., 2019), outcomes in high school were examined separately by intervention, cohort, and sex. Biological sex was examined as a grouping variable because other dating violence interventions have found differential effects for males and females (Foshee et al., 2004a, b; Wolfe et al., 2009), and cohorts were examined separately because of the possibility that implementation timing impacted effectiveness.

The longitudinal multiple group modeling framework (LGM (Little et al., 2021), which imposes parsimony on complex models through considered placement of equality constraints) has been recommended as a promising approach to the problem of evaluating program effects across many time points, groups, and/or outcomes while controlling the risk of capitalizing on chance (Little et al., 2021). For each outcome in this study, program effects were evaluated comparing DM and SC means across sex and cohort at each timepoint (11th grade timepoint was only evaluated in Cohort 3) for a total of 20 independent means per model. The LGM framework provides a method of reducing the total number of estimated means by identifying sets of means with similar values and placing equality constraints on each set to test the assumption that the means are statistically indistinguishable. Guided by the freely estimated means, as well as the main hypotheses (DM exposure reduces students’ risk for violence and increases relationship skills) and a set of secondary guidelines (e.g., favor constraints that minimize gender and cohort differences, where possible), we used the LGM framework to estimate a reduced number of means, halting the simplification process when constraints resulted in poor overall model fit*.*^3^ In the resulting simplified model, means sharing the same equality constraint are assumed to be statistically indistinguishable, while means not sharing a constraint are assumed to be statistically distinct.

From the final model parameters, we calculated the relative risk ratio (RRR), which represents the reduction in risk seen in the DM students relative to their SC counterparts. For consistency, we scaled the RRR for Positive Relationship Skills to represent risk (lack of skills). Because statistically indistinguishable group means are constrained to be equal, some RRRs may be identical across sex and/or cohort.

## Results

### TDVP

**Fig. 2:**
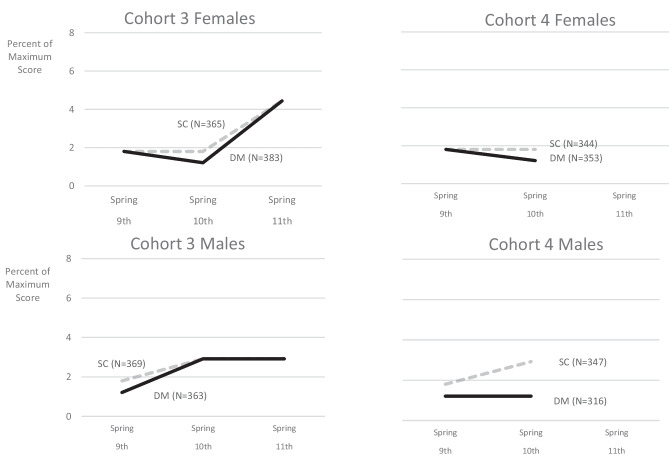
Final models demonstrating intervention effects for teen dating violence perpetration across time by sex and cohort. Note. SC= Standard of Care condition. DM= Dating Matters condition. Percent of Maximum Score (POMS) refers to the maximum possible score given the number of items and response categories in a scale, rather than the maximum observed score. In the final models, significant differences between DM and SC are represented by non-overlapping lines, where non-significant differences were constrained to be equal without substantially decreasing model fit

By spring of Grade 9, program effects for TDVP were found for males in both cohorts but not for females (RRR = 32.78, 95% CI = 15.55 to 50.01).^4^ By spring of Grade 10, program effects were found for females in both cohorts (RRR = 32.78, 95% CI = 15.55 to 50.01) and for males only in Cohort 4 (RRR = 58.56, 95% CI = 47.80 to 69.32). By spring of Grade 11, no TDVP program effects were found for Cohort 3 students (see eTable 4 and Fig. 2).

### TDVV

**Fig. 3:**
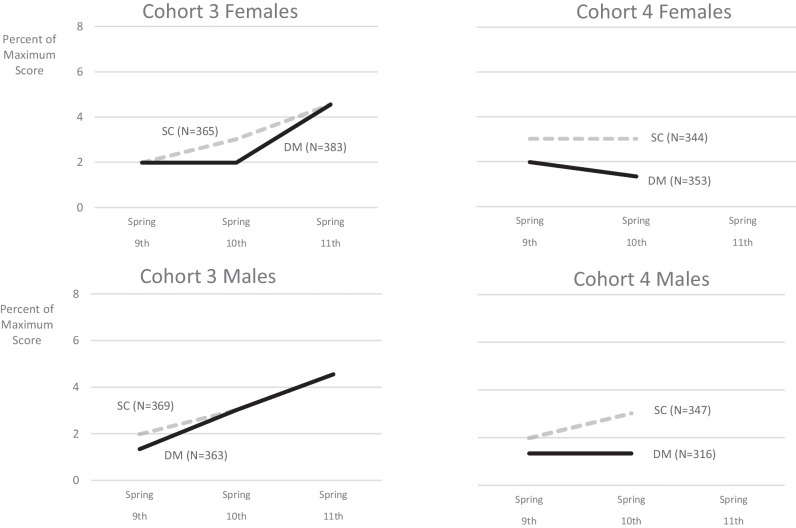
Final models demonstrating intervention effects for teen dating violence victimization across time by sex and cohort. Note. SC= Standard of Care condition. DM= Dating Matters condition. Percent of Maximum Score (POMS) refers to the maximum possible score given the number of items and response categories in a scale, rather than the maximum observed score. In the final models, significant differences between DM and SC are represented by non-overlapping lines, where non-significant differences were constrained to be equal without substantially decreasing model fit

By spring of Grade 9, significant program effects were found for females in Cohort 4 (RRR = 34.44, 95% CI = 24.15 to 44.73) and for males in both cohorts (RRR = 32.32, 95% CI = 11.88 to 52.77). By spring of Grade 10, significant program effects were found for females in Cohort 3 (RRR = 34.44, 95% CI = 24.15 to 44.73) and for both males and females in Cohort 4 (RRR = 55.63, 95% CI = 42.67 to 68.58). By spring of Grade 11, no TDVV program effects were found for Cohort 3 students (see eTable 5 and Fig. 3).

### NCRS

**Fig. 4:**
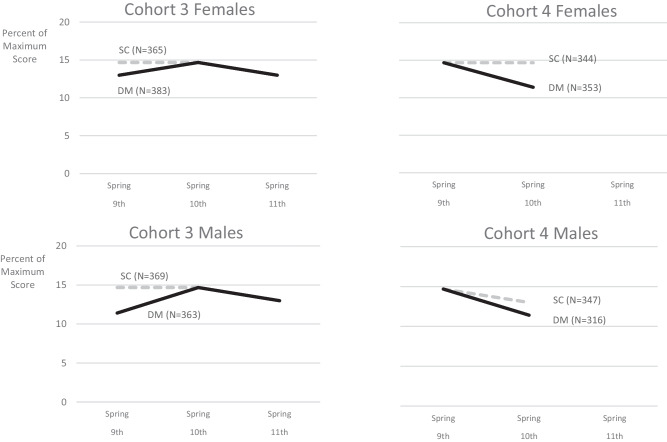
Final models demonstrating intervention effects for negative conflict resolution strategies across time by sex and cohort. Note. SC= Standard of Care condition. DM= Dating Matters condition. Percent of Maximum Score (POMS) refers to the maximum possible score given the number of items and response categories in a scale, rather than the maximum observed score. In the final models, significant differences between DM and SC are represented by non-overlapping lines, where non-significant differences were constrained to be equal without substantially decreasing model fit

By spring of Grade 9, significant program effects were found for females and males in Cohort 3 only (females, RRR = 11.45, 95% CI = 6.32 to 16.59; males, RRR = 22.29, 95% CI = 16.19 to 28.39). By spring of Grade 10, program effects were evident for females and males in Cohort 4 only (females, RRR = 22.29, 95% CI = 16.19 to 28.39; males, RRR = 12.24, 95% CI = 4.54 to 19.94). No NCRS program effects were found for Cohort 3 students by spring of Grade 11 (see eTable 6 and Fig. 4).

## PRS

**Fig. 5:**
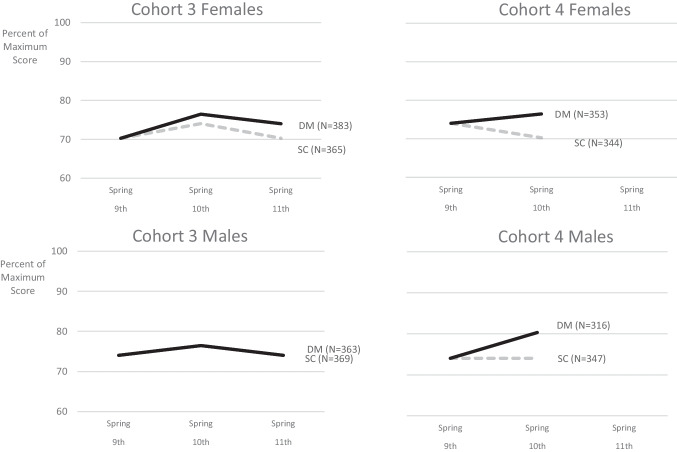
Final models demonstrating intervention effects for positive relationship skills across time by sex and cohort. Note. SC= Standard of Care condition. DM= Dating Matters condition. Percent of Maximum Score (POMS) refers to the maximum possible score given the number of items and response categories in a scale, rather than the maximum observed score. In the final models, significant differences between DM and SC are represented by non-overlapping lines, where non-significant differences were constrained to be equal without substantially decreasing model fit

No program effects were evident by spring of Grade 9, but by spring of Grade 10, program effects were found for females in Cohort 3 (RRR = 9.36, 95% CI = 3.71 to 15.00) and females and males in Cohort 4 (females, RRR = 20.82, 95% CI = 15.27 to 26.37; males, RRR = 24.18, 95% CI = 13.60 to 34.77). By spring of Grade 11, females in Cohort 3 showed program effects (RRR = 12.65, 95% CI = 8.26 to 17.04) (see eTable 7 and Fig. 5).

### Summary of Findings

**Fig. 6:**
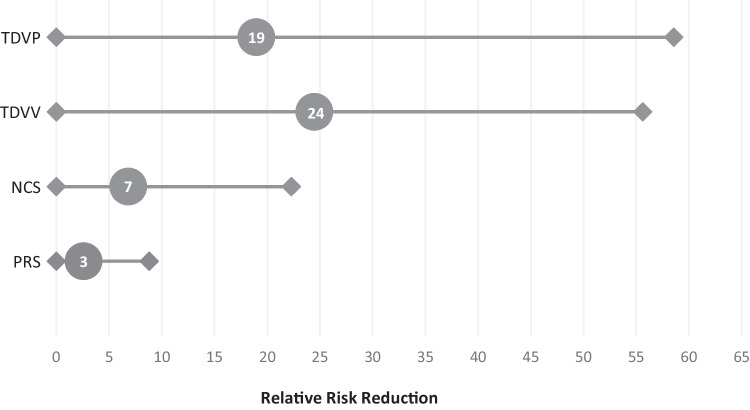
Minimum, mean, and maximum relative risk reduction for Dating Matters® (DM) vs. standard of care (SC), aggregated by cohort, sex, and time periods. Note: Relative risk reduction represents a ratio of Dating Matters (DM) to standard-of-care (SC) means. Values less than 100 indicate a reduction of risk (e.g., 19 = 19% reduction). TDVP = teen dating violence perpetration. TDVV = teen dating violence victimization. NCRS = negative conflict resolution strategies. PRS = positive relationship skills

Table 1 summarizes the significant program effects for each outcome by assessment grade (9, 10, 11), cohort (3 or 4), and sex (males and females). Figure 6 presents a summary of the RRR across all four outcomes. On average across groups and time, students in schools participating in DM had a 13% lower risk across all outcomes, including TDVP and TDVV, NCRS, and (lack of) PRS, in their high school dating relationships than did students in comparison schools who received prevention programming in 8th grade only. On the primary outcomes specifically, DM students had 19% and 24% lower risk, on average, for TDVP and TDVV during high school, respectively, compared to SOC. Effects of the DM program on PRS and NCRS were less strong with 7% and 3% average risk reductions, respectively.

**Table 1:** Summary of evidence supporting hypothesized program effects

	**Grade 9**				**Grade 10**				**Grade 11**	
	**Cohort 3**		**Cohort 4**		**Cohort 3**		**Cohort 4**		**Cohort 3**	
	**Males**	**Females**	**Males**	**Females**	**Males**	**Females**	**Males**	**Females**	**Males**	**Females**
**Teen dating violence perpetration (TDVP)**	✓		✓			✓	✓	✓		
**Teen dating violence victimization (TDVV)**	✓		✓	✓		✓	✓	✓		
**Negative conflict resolution strategies (NCRS)**		✓		✓		✓		✓		
**Positive relationship skills (PRS)**						✓		✓		✓

Checkmarks indicate findings that support the hypothesis. Blank cells indicate null effects (DM and SC are not significantly different)

## Discussion

This study examined the longitudinal effects of the *Dating Matters* (DM) middle school intervention on several TDV-related outcomes in high school. Findings suggest that, on average, the *Dating Matters* comprehensive prevention model, implemented in middle school, continued to be more effective at reducing the risk of TDV perpetration, TDV victimization, and use of negative conflict resolution strategies in high school than an evidence-based comparison program implemented in 8th grade only, although the results were less consistent across cohort and sex than was true in the middle school evaluation. Results for teen dating violence perpetration, teen dating violence victimization, and negative conflict resolution skills extend the short-term results that were found in middle school (Niolon et al., 2019). However, although no differences were found during middle school on the use of positive relationship skills, significant program effects for this outcome were found in 10th and 11th grades. These findings demonstrate long-term impacts of *Dating Matters* on our primary outcomes of interest. Although the patterns of these effects were not consistent across groups (i.e., sex, cohort, grade), positive effects were found for both cohorts and sexes on all outcomes (see Fig. 6 for a summary of relative risk reductions for all four outcomes). In general, more significant effects were found among Cohort 4 students than among Cohort 3 students, which is notable considering that Cohort 4 was only followed through 10th grade while Cohort 3 was followed to 11th grade. Overall, significant effects were found for both cohorts, for males and females, and across grades 9 and 10. Only Cohort 3 was assessed in 11th grade, but only one significant effect was found in grade 11 (i.e., PRS for females only), indicating that effects of the program may be waning over time.

For TDV perpetration, significant program effects were found for both cohorts of males in 9th grade and one cohort of males in 10th grade and for both cohorts of females in 10th grade. It is not clear why effects on TDV perpetration were only detected for males in 9th grade, while the effect for females did not emerge until 10th grade. However, the fact that 3 of the 4 groups demonstrated significant program effects in 10th grade on perpetration of TDV following an intervention that ended in 8th grade is noteworthy. Significant average relative risk reductions for TDV perpetration ranged from 33 to 59%, with an average of 19% across all waves and groups (this average includes both significant and nonsignificant effects); the sizes of these average relative risk reductions are also notable considering that *Dating Matters* was being compared to an evidence-based TDV prevention program delivered in 8th grade. However, the lack of effects in 11th grade suggests that the long-term impacts of the intervention may wane over time, and booster or follow-up programming in high school might be helpful in continuing the effects on TDV perpetration into late adolescence and young adulthood.

For TDV victimization, significant program effects were found for both cohorts of males in 9th grade and one in 10th grade; effects were found for both cohorts of females in 10th grade and one in 9th grade. Overall, the effects suggest the middle school intervention was successful in reducing TDV victimization into high school; this finding is particularly notable given that many of the middle schools in our study did not feed into one high school but sent students to many different high schools, meaning that many of the students in our sample went to high school with peers who had not received Dating Matters in middle school. Significant average relative risk reductions for TDV victimization ranged from 32 to 56%, with an average of 24% risk reduction for DM students compared to SC students across all waves and groups. As with TDV perpetration, no significant program effects for TDV victimization were found for Cohort 3 in 11th grade, indicating the potential for waning effects over time.

For use of negative conflict resolution strategies, a different pattern emerged. Effects were found for Cohort 3 in 9th grade only and for Cohort 4 in 10th grade only. Although potential reasons for this pattern are unclear, the findings suggest that *Dating Matters* also continued to reduce the use of negative conflict resolution strategies in dating relationships into high school. Significant average relative risk reductions for use of negative conflict resolution strategies were smaller than those for TDV perpetration and victimization, ranging from 11 to 22% with an overall average of 7% risk reduction for DM students compared to SC students across all waves and groups. As with TDV perpetration and victimization, no effects were detected in Cohort 3 in 11th grade for either male or female students.

Significant program effects were found for the use of positive relationship skills for all groups in 10th grade except for Cohort 3 males and for Cohort 3 females only in 11th grade. These effects are particularly interesting given that no effects were found on this outcome in middle school, where sizeable ceiling effects were identified (Niolon et al., 2019). Increased use of positive relationship skills may reflect the developmental trajectory toward having more serious romantic relationships as youth age, providing additional opportunities to use relationship skills gained in the *Dating Matters* programs, making detection of these effects more likely. Significant average relative risk reductions for lack of^5^ positive relationship skills were smaller than those for TDV perpetration and victimization, ranging from 9 to 24% with an overall average risk reduction of 3% for DM relative to SC students across all waves and groups.

Overall, a slightly higher number of significant program effects were detected in Cohort 4 than in Cohort 3 (11 significant effects v. 7 in 9th and 10th grades; see Table 1), and it is possible that Cohort 4, having started *Dating Matters* in 6th grade during the *second* year of implementation (as opposed to starting 6th grade in the *first* year of implementation as with Cohort 3), may have received the *Dating Matters* components delivered with better fidelity to the intended intervention, especially in their first (6th grade) year. It is difficult to determine whether this is the case; anecdotally, we know that sites improved in their administration of all components of *Dating Matters* after their first year and in each subsequent year and Cohort 4 demonstrated better program effects on some of these outcomes in middle school as well (Niolon et al., 2019).

In sum, evidence of continued effectiveness through high school for this middle school intervention, particularly on TDV outcomes, is promising and adds to existing evidence on the potential for creating sustained change in risk for dating and intimate partner violence through early prevention efforts. These findings add *Dating Matters* to the relatively short list of TDV prevention programs that have sustained effects beyond the end of program implementation (Foshee et al., 2004a, b; Wolfe et al., 2009). Evaluations of other adolescent risk behavior interventions have found that when effects on primary and secondary outcomes are sustained beyond the intervention, the effect sizes tend to be small and often fade over time (Ellickson et al., 1993; Hale et al., 2014). Despite the relatively robust findings and relative risk reductions in 9th and 10th grade, the lack of significant findings in 11th grade in the one cohort with available data suggests that *Dating Matters* may be similar to other interventions in terms of waning effects and that implementation during middle school alone may not be sufficient to sustain significant risk reduction through later adolescence. It is notable that *Dating Matters* was compared to an existing evidence-based intervention, *Safe Dates*, in this comparative effectiveness trial—thus, any effects measured were those that were above and beyond the anticipated effects of *Safe Dates*. *Safe Dates*, implemented in 8th grade in both conditions of this RCT, has previously been found to have long-term effects on teen dating violence in a four-year follow-up through high school (Foshee et al., 2004a, b). Overall, the current findings suggest the need for additional violence prevention efforts in high school to boost and extend skills developed through middle school intervention. Further, to achieve primary prevention, *Dating Matters* was intentionally designed to be delivered in middle school, before young people typically engage in the more intimate romantic relationships that are common in later adolescence. However, it is possible that older adolescents may need additional education and opportunities for skill-building in high school prior to the drop-off in effects observed in 11th grade. It should be considered that the effects observed in middle school and the earlier years of high school may translate into reduced risk for other outcomes in later adolescence and adulthood; for example, prevented TDV victimization in 9th grade could mean reduced sexual risk behavior or reduced mental health problems in late adolescence, even if effects on TDV victimization wane by 11th grade. Further research could examine such associations.

### Limitations

In addition to the limitations of the overall RCT described elsewhere (Niolon et al., 2019) the high school follow-up of this RCT has several limitations. First, our data collection infrastructure did not enable us to follow each of the two full-exposure cohorts (3 & 4) through the end of high school or into young adulthood. We could not assess Cohort 4 beyond 10th grade, which limits our ability to truly understand the trajectory of the effects of *Dating Matters* in the longer term. Second, this was a comparative effectiveness trial without a no-treatment comparison group; it is impossible to know the true extent and size of the effects of *Dating Matters* relative to no intervention. Third, we decided only to include schools in the final analysis who had participated in our trial for at least two years, in part because schools who did not participate for two years had implemented less than half of the Dating Matters intervention and the students had completed less than half (in some cases only the baseline) of the data collection assessments; however, this decision meant that we were not able to utilize a strict intent-to-treat design, when usually data collection continues even if the intervention is not completed. Fourth, given the flexibility of the LGM framework, different analysts might produce minor variations in the selection of constraints, even when using the same guiding hypotheses and assumptions. For this reason, it is best to conduct replication studies designed to probe and verify specific effects (e.g., a given group at a given time point).

We can say, given the overall results, that there is persuasive evidence for beneficial effects of participation in the *Dating Matters* comprehensive model on teens’ relationship behaviors in high school. More research is needed to understand the mechanisms of impact for different students at different times in their development.

## Conclusions

The findings from this study highlight the benefit of early and comprehensive TDV prevention efforts that address risk and protective factors among youth but also with their parents or caregivers, schools, and communities. Our results, overall, suggest that this multi-year, comprehensive prevention model is more effective than the single-year, single program model to which we compared it in, at least, the short and medium term and may help set the stage for long-term risk reductions through adulthood, especially if combined with additional prevention approaches through early adulthood. Further research should examine component-specific effects to help determine whether all components are necessary to maintain program effects and to examine mechanisms of change where program effects exist. Implementation research should investigate the feasibility of implementing *Dating Matters* with fidelity in the field, given the resources necessary to implement its multiple components and multi-year design. Prior research with *Dating Matters* also points to the potential for effectiveness beyond the primary intended outcomes of TDV and relationship behaviors to other forms of violence and risk behavior (DeGue et al., 2021; Estefan et al., 2021; Vivolo-Kantor et al., 2021); additional research should assess whether those effects are sustained as well. The identification of effective comprehensive prevention models that address cross-cutting risk and protective factors for violence, such as healthy communication skills and social-emotional development, can reduce the burden on communities to implement multiple prevention strategies addressing different but related health outcomes. Early comprehensive prevention efforts can improve the overall health and well-being of youth, and their communities, throughout their lifetimes.

### Supplementary Information

Below is the link to the electronic supplementary material.

Supplementary file1 (DOCX 371 KB)

## Footnotes

1 See Niolon et al. (2019) for more details on the model components and background*. Dating Matters* is available at https://vetoviolence.cdc.gov/apps/dating-matters-toolkit.

2 Number of days between the student’s birthdate and November 22 of each student’s Grade 6 year. This can be interpreted as the student’s age, relative to his/her grade mates, regardless of cohort.

3 Model fit was poor when a chi-square difference test against a model with freely estimated means resulted in a *p* value of less than .20.

4 Due to equality constraints on parameters, effect sizes across cohort or sex can be identical. When one RRR is provided for two significant effects, those estimates were identical.

5 The use of positive relationship skills in a dating relationship was reverse coded for analysis so effects are consistently interpreted as reduction in risk.
