# Correction: High School Follow-Up of the Dating Matters^®^ RCT: Effects on Teen Dating Violence and Relationship Behaviors

**DOI:** 10.1007/s11121-024-01691-w

**Published:** 2024-05-28

**Authors:** Phyllis Holditch Niolon, Lianne F. Estefan, Sarah DeGue, Vi D. Le, Allison J. Tracy, Colleen Ray, Daniel Bontempo, Todd D. Little, Alana M. Vivolo-Kantor, Natasha Latzman, Bruce Taylor, Andra Tharp

**Affiliations:** 1 Division of Violence Prevention, National Center for Injury Prevention and Control, Centers for Disease Control and Prevention, 4770 Buford Highway NE, Atlanta, GA30341, S106-10 USA; 2 TJFACT Inc, Contractor for the Division of Violence Prevention, Atlanta, GA USA; 3 College of Education, Texas Tech University, Lubbock, TX USA; 4 North-West University, Vanderbijlpark, South Africa; 5 Division of Overdose Prevention, National Center for Injury Prevention and Control, Centers for Disease Control and Prevention, Atlanta, GA USA; 6 Harvard, Massachusetts, MA USA; 7 NORC, University of Chicago, Chicago, IL USA; 8 Sexual Assault Prevention and Research Office, Department of Defense, Washington, DC USA

**Correction to: Prevention Science (2024) 25:603–615** 10.1007/s11121-024-01648-z

The article “High School Follow-Up of the Dating Matters^®^ RCT: Effects on Teen Dating Violence and Relationship Behaviors”, written by Niolon, P.H., Estefan, L.F., DeGue, S., Le, V.D., Tracy, A.J., Ray, C., Bontempo, D., Little, T.D., Vivolo-Kantor, A.M., Latzman, N., Taylor, B., and Tharp, A., was originally published Online First without Open Access. After publication in volume 25, issue 4, page 603–615 the author decided to opt for Open Choice and to make the article an Open Access publication. Therefore, the copyright of the article has been changed to © The Author(s) 2024 and the article is forthwith distributed under the terms of the Creative Commons Attribution 4.0 International License, which permits use, sharing, adaptation, distribution and reproduction in any medium or format, as long as you give appropriate credit to the original author(s) and the source, provide a link to the Creative Commons licence, and indicate if changes were made. The images or other third party material in this article are included in the article’s Creative Commons licence, unless indicated otherwise in a credit line to the material. If material is not included in the article’s Creative Commons licence and your intended use is not permitted by statutory regulation or exceeds the permitted use, you will need to obtain permission directly from the copyright holder. To view a copy of this licence, visit http://creativecommons.org/licenses/by/4.0/.

The original article has been corrected.

