# Supplementary material for: High School Follow-Up of the Dating Matters® RCT: Effects on Teen Dating Violence and Relationship Behaviors
Source: Prev Sci. 2024 Mar 8;25(4):603–15. doi: 10.1007/s11121-024-01648-z (PMC11111327; doi:10.1007/s11121-024-01648-z)
Supplement: Supplementary file 1 — Supplementary file1 (DOCX 371 KB) [file 11121_2024_1648_MOESM1_ESM.docx]

SUPPLEMENTAL ONLINE METHOD

High School Follow-up of the Dating Matters^®^ RCT: Effects on Teen Dating Violence and Relationship Behaviors

This online supplement contains supplemental text, tables, and a figure to accompany the manuscript describing the high school follow up of the RCT evaluating Dating Matters’ effects on primary outcomes of the trial. The text contains additional detailed information on the components of the Dating Matters model, the randomization process for the RCT, the process for handling and imputing missing data, and covariate and analysis strategies.

Program components:

The following graphic, depicting the program components of the Dating Matters comprehensive prevention model, was retrieved from [www.cdc.gov/violenceprevention/intimatepartnerviolence/datingmatters/about.html](http://www.cdc.gov/violenceprevention/intimatepartnerviolence/datingmatters/about.html) on October 3, 2023


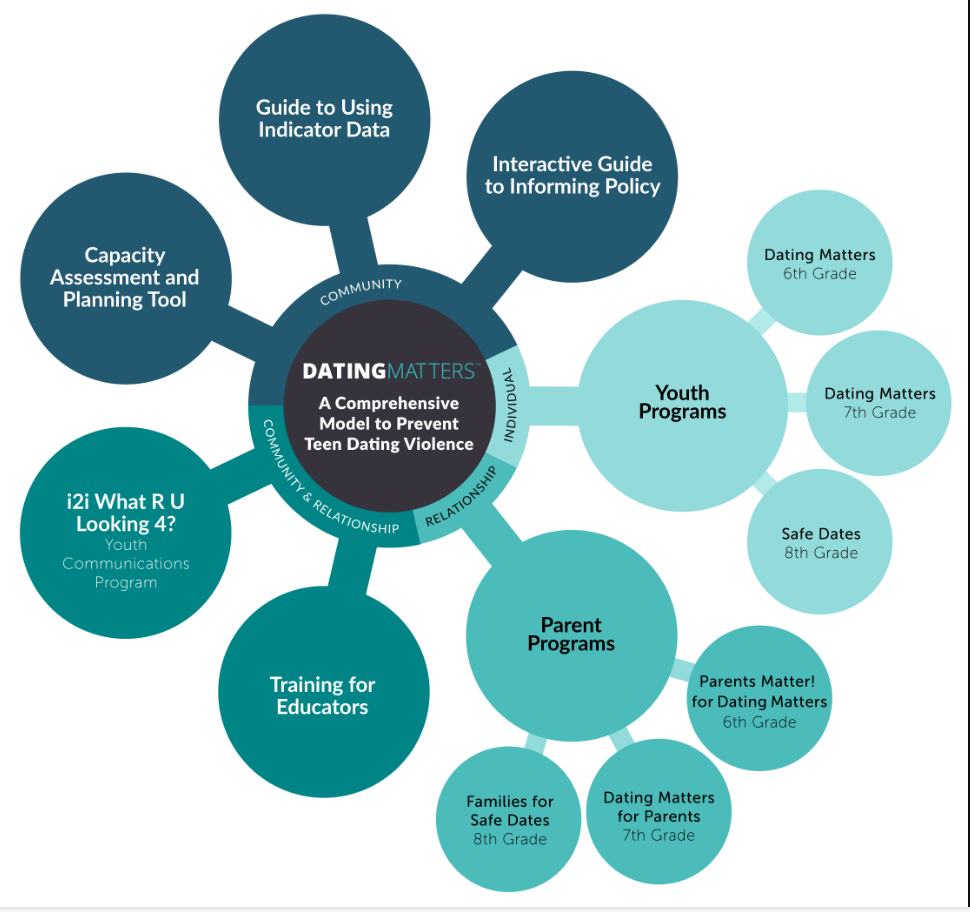


These components are described in great detail in the eMethods of Niolon et al., 2019. The following includes a quoted excerpt from the eMethods of Niolon et al., 2019:

“The *Dating Matters* model^8,9^ includes the following interventions:

Youth programs. The 6^th^ and 7^th^ grade youth programs were developed by CDC. Eighth grade students received *Safe Dates*,^10^ an evidence-based dating violence prevention program. All three curricula use social-emotional learning and skills-based approaches to focus on healthy relationships and help youth learn and practice healthy relationship skills, such as communication and conflict resolution. The 6^th^ grade curriculum includes 6 classroom sessions focusing on healthy relationships broadly, while the 7^th^ grade (7 classroom sessions) and 8^th^ grade curriculum (10 classroom sessions, a poster contest, and a play) are more focused on dating relationships. DM also includes an integrated, youth-delivered and youth-focused communications program, *i2i: What R U Looking 4,* designed to reinforce messaging from the DM curricula for all grades through a near-peer brand ambassador program, in-person activities, youth-developed materials, and digital resources led by DM brand ambassadors, who are volunteer high school students from the same neighborhoods as students in the DM middle schools.

Parent programs. In the DM condition, parents of 6^th^ graders received a CDC-adapted version of the *Parents Matter!* program, an evidence-based program for decreasing sexual risk and promoting positive parent-child communication about sexual health among adolescents.^11^ Parents of 7^th^ graders received a parent training program developed by CDC specifically for DM that was focused on positive parenting and parent-child communication. Parents of 8^th^ graders received *Families for Safe Dates*, an evidence-based dating violence prevention intervention.^12^ Parent training was provided in community-based settings and not offered to parents of youth in the SC condition.

School and community-level interventions. All educators in DM schools were asked to complete an online training developed by CDC to provide educators and school personnel with TDV knowledge and resources and to motivate them to implement prevention activities in their schools. Local health departments implementing DM also engaged in a capacity and readiness assessment using a CDC-developed tool called the *Dating Matters Capacity and Readiness Tool* (DM-CAPT), as well as activities at the community-level aimed at tracking and informing local policy and data use related TDV prevention; these activities may have impacted students in both the DM and SC schools. See eTable 2 for further depiction of the DM intervention compared to the SC condition. See [www.cdc.gov/violenceprevention/datingmatters](http://www.cdc.gov/violenceprevention/datingmatters) for more information on the DM component interventions.”

Randomization

Block randomization was considered but not ultimately conducted because schools within each site were demographically similar. After recruiting a quota of middle schools in each site, an independent team contracted for data collection (NORC) conducted simple random assignment of schools to either the DM or the SC condition within each of the four sites, using a random number generator. NORC and CDC jointly explained the randomization process to the sites and informed school administrators and staff of the school’s assigned condition. As shown in the CONSORT diagram (Figure 1, main manuscript), some schools subsequently dropped from the study. Additional schools that met eligibility requirements were admitted to the study on a rolling basis, receiving a simple random assignment to condition after agreeing to participate.

Missing Data

There was substantial missing data stemming from both planned and unplanned sources. Planned missing data was due to the data collection and survey design.^1^

- Students in certain grade and cohort combinations were not assessed in our cohort sequential design.
- In high school, sampling was instituted to decrease the study’s cost.
- The baseline survey version, which assessed lifetime occurrence of some behaviors, was designed to be administered to students in Grade 6. In newly participating schools, however, *all students* were administered the baseline survey version in the fall assessment, including students in Grades 7 or 8. For these older students, lifetime responses could not be combined with 4-month responses given by other Grade 7 and 8 students in schools continuing participation in the study. Therefore, we treated the responses arising from wording differences across the two survey versions as distinct variables, each with its own missing data pattern. This approach differs from our previous approach in the middle school evaluation studies,^2-5^ which addressed item wording differences through covariate adjustment.
- Some items were part of a skip-and-fill pattern. For example, the teen dating violence outcome measure was asked only of students who indicated in the same survey that they had dated.

Unplanned missing data arose from the following sources.

- Time-varying guardianship status in high school was impacted by a temporary misalignment of response option wording with radio buttons in the online survey before the problem was identified and corrected. Affected responses were set to missing.
- Some schools entered the study late or exited early.
- Individual students joined or left participating schools.
- Students had the option to refuse to respond to items within the survey.

Imputation Procedures

Prior to empirical imputation, we hard coded some types of missing data using logic. For example, students who attended a participating school but who were not present during survey administration were given a survey date derived from the date (or average of dates) for the associated wave of data collection at their school.

Multiple imputation (MI) is commonly used to handle missing data in survey research and epidemiological studies.^6-8^ Multiple imputation by chained equations (MICE), also known as fully conditional specification (FCS), was applied to an inclusive imputation model containing student survey data from all nine occasions and some school-level descriptive data (e.g., proportion of the student body receiving free or reduced school lunch). Using related items at the same occasion and the same item at other waves, in addition to demographics and age/timing data, increases accuracy of the conditional expectations for imputed placeholder values under a missing at random (MAR) mechanism.

The application of MI for these data can be described as a general imputation strategy on an ultra-inclusive imputation dataset. A general imputation strategy addresses many outcomes and covariates and permits multiple analyses using selected variables from the imputed datasets. This approach is contrasted with an imputation containing fewer variables that is targeted for use with a single analysis model.^[[1]](#footnote-2)^ For complex and challenging imputations, especially where the imputer and analyst may differ, a general strategy might be preferred. Adopting a general strategy raises computational issues that increase with the size of the imputation dataset and can result in memory and speed limitations on computation. With FCS, each outcome is conditioned on every other outcome. Consequently, the models for each outcome can contain more predictors than cases, predictors with problematic multicollinearity, many predictors with little relation to the outcome, or other circumstances that degrade model estimation.^[[2]](#footnote-3)^ Collins and colleauges^9^ provide evidence supporting greater inclusivity because they show no harm when including additional predictor/auxiliary variables that have no association with the outcome. The methodology of principal components as auxiliary variables^10^ renders ultra-inclusive imputation datasets more computationally tractable in two important ways. First, the “signal” in the dataset is captured in a parsimonious set of component scores obtained with principal component analysis (PCA) (retained linear components). Second, multicollinearity issues are eliminated, as components are orthogonal. As implemented in the *R* package *PcAux*, additional automation and resource-sparing features are available.^11^

One of the most important automations delivered by *PcAux* relates to interaction variables. All non-linear transformations of variables, including interactions, to be used in any subsequent analysis model must also be included in the imputation model.^12^ With a general imputation strategy, where a general set of interaction terms must be provided, #var (#var - 1)/2 interaction terms must be computed and added to the imputation dataset to cover all possible two-way interactions. With the *PcAux* software, only #var * #comp variables need be added,^[[3]](#footnote-4)^ where #comp is the number of retained linear components. Additionally, *PcAux* provides the option to further reduce the number of interaction terms to #mod * #comp variables, where #mod is a parsimonious user-specified list of moderators used to form two-way interaction variables.

Using all nine waves of survey data,^[[4]](#footnote-5)^ we used the implementation of MICE available in the *R* package (*mice* v3.12.0).^13,14^ As mentioned, imputation procedures were optimized and automated by the method of principal components as auxiliary variables using the *PcAux* software^[[5]](#footnote-6)^ to facilitate imputation of these data.^10^ The models in the MICE chain were set to predictive-mean-matching (pmm) for continuous, ordinal, and binary variables.^[[6]](#footnote-7)^ Polytomous regression (polR) was used for nominal variables. There were 3053 variables in the incomplete dataset. Many variables were items on multi-item scales assessed at each of nine waves. After data reduction with PCA, 863 linear components explaining 85% of dataset variance were retained. For the longitudinal multi-group analysis models (LMG) used in this paper, the grouping variables are moderators when parameters are unconstrained across groups. Consequently, it was necessary that, at a minimum, all two-way interactions of analysis and grouping variables be included in the imputation dataset. The full set of moderators used for the general imputation of these data were (1) age at Grade 6, (2) cohort, (3) race, (4) treatment condition, (5) gender, and (6) site,^[[7]](#footnote-8)^ yielding a nonlinear dataset of nine moderator variables multiplied by the 863 linear components yields 7767 interaction terms. After a second PCA, 689 additional components explaining 35% of the nonlinear dataset variance were retained, designated as nonlinear component scores. One hundred imputations were generated using only the 863 linear and 689 nonlinear (totaling 1552) *PcAux*-derived component scores as potential predictors. For each model in the MICE chain, components with less than r = 0.10 association with the model outcome were excluded from the model’s predictor set.

After generating the imputations, the quality of the imputed placeholder values was examined with diagnostic plots juxtaposing the density profile of observed and imputed values.^15^ Discrepancies in density profiles were generally small and plausible under a MAR mechanism. These imputed datasets support the use of a general set of two-way interactions in analysis models, and specifically support the multi-group structural equation models (SEM) used in this paper.

Equivalence Evaluation

We evaluated the pairs of *Dating Matters* (DM)/standard-of-care (SC) groups within gender, cohort and wave for potential nonequivalence with respect to baseline values of outcome variables and student demographic information and other characteristics (eTable 3). This equivalency evaluation revealed the need to adjust the group means for imbalances.

**Covariate Measures**

Five types of variables were constructed for covariate adjustment: (1) weighted effects-coded variables representing race,^[[8]](#footnote-9)^ guardianship,^[[9]](#footnote-10)^ and site,^[[10]](#footnote-11)^ (2) dichotomous indicators representing survey mode,^[[11]](#footnote-12)^ and witnessing violence,^[[12]](#footnote-13)^ (3) date-anchored variables representing relative age and assessment timing,^[[13]](#footnote-14)^ and (4) baseline outcome scores, rescaled to have a minimum value of zero. These variables retain equivalent zero-points across groups and time to maintain standardized percent of maximum score (POMS) units for comparison. In other words, no variable was centered on the group/time-specific mean.

Covariate Adjustment Strategy

Before covariate adjustment, we rescaled the parcel scores using POMS to simplify interpretation of program effect sizes. POMS rescaling involves subtracting 1 (lowest response) from the item average to obtain a zero-anchor (min = 0 POMS) and then dividing by the number of response options minus 1 to obtain scores that range from 0 to 1 [e.g.., (1 - 1)/(4 - 1)=0; (4 - 1)/(4 - 1) = 1]. We then multiplied by 100 to obtain percentage of the maximum score possible (max = 100 POMS).

To simplify the already complex program evaluation models, we conducted the covariate adjustment *before* fitting the evaluation models. Because parcels were to be used as indicators of a common latent variable, we considered the shared variance rather than the total variance of the parcels to be critical. Therefore, we stacked the data by parcel to approximate the covariate adjustment at the latent construct level. We created dummy codes to identify the second and third parcel in the parcel-stacked data. These parameters allow covariate-adjusted scores to be calculated uniquely for each parcel from the parameters of the model.

We conducted the covariate adjustment separately by cohort, gender, treatment, and wave. This disaggregated approach provides for both main and interaction effects of covariates^17^ but risks overfitting the sample data. Within these smaller subgroups, there was reduced variability in some parcel indicators, putting covariate estimates at possible risk of being overestimated (e.g., due to an influential outlier) or underestimated (e.g., due to restricted variability). To minimize these risks, we first ran the covariate adjustment models and output the residuals for all students. We then identified students with residuals in the upper and lower 2.5% tails of the distribution. The covariate adjustment model was re-run on a subsample in which the outlying students were omitted.

Covariate-adjusted scores were calculated from the model results in the following manner. First, residuals were calculated for all students, including students which were omitted from the trimmed covariate models, ^[[14]](#footnote-15)^ using predicted scores (Y-hat) derived from parameters estimated in the trimmed models subtracted from the preadjusted parcel scores. Since residuals have a theoretical center at zero, the POMS minimum zero point was restored by adding the intercept for Parcel 1 or the intercept plus the regression coefficient of the associated dummy-coded indictor for Parcel 2 or Parcel 3. The resulting covariate-adjusted outcome scores are sample-representative regarding age, race/ethnicity composition, and guardian status composition. In addition, we calculated the adjusted scores to reflect students who had not witnessed violence, who completed the survey at the target assessment date (March 28), and who took the survey online. The comprehensive list of covariates was included to ensure that the program effects were not confounded with any of the control variables. Moreover, the number of variables needed to represent these potential confounders was simply too many to include in the analysis phase. Therefore, we chose a two-stage approach whereby the covariate control regressions were done on the parceled indicators of the constructs. We then took these covariate-adjusted indicators and used them in the analysis models.

Latent Variable Models

Indicated by three parcels for each outcome, the latent variables were estimated using effects-coded latent variable scaling constraints to retain the POMS metric of the indicators.^18^ We imposed measurement invariance on these loading and intercept parameters across wave and group. All latent variable models were fit using the Mplus statistical program (Version 8.5).^19^

Because the study ended after Grade 10 for Cohort 4 students, we discarded imputed Grade 11 values and created random normal values and making them orthogonal to all other variables in the analysis, using regression residualization. This step satisfies the structural requirement of a parcel-indicated latent construct at Grade 11 in the Cohort 4 groups but avoids any contribution to global model misfit that chance correlation among parcels calculated from imputed values may have introduced. There were no constraints or hypothesis testing involving the Grade 11 values for Cohort 4.

Strategy for Evaluation Model Testing

We fit longitudinal multiple group models (LMG) with equality constraints on the group means to evaluate program effects.^20^ When there are many points of evaluation (many waves, many groups), uncorrected tests of individual program effects may inflate the possibility of false positives (Type I errors). Traditional corrections, such as Bonferroni corrections or reliance on an omnibus test, may err in the opposite direction, leading to a failure to detect true program effects (Type II errors). The LMG framework allows for an evaluation of all DM/SC pairs, but due to equality constraints across parameters, each is no longer independent, presenting a lower risk of capitalizing on chance variation in the data. Multiple testing bias motivated our use of the LGM framework. This novel framework provides a way to preserve important nuances while reducing the overall number of hypothesis tests. Constraints imply that some DM/SC comparisons (program effects) are identical, thereby reducing the number of tests overall.

One strength of the LGM framework is the flexibility with which constraints can be placed. The selection of parameters to include in a given equality constraint relies on a process that attempts to inform empirical similarity of the freely estimated parameters with theoretical expectations. In other words, the LGM framework intentionally overlays subjective assumptions (e.g., that there are no cohort differences) on empirical patterns to explicitly test those assumptions. If the model is notably degraded when subjective assumptions are imposed, those assumptions are faulty; if the model fit is essentially unchanged, the assumptions are allowed to stand, therefore simplifying the conclusive statements that can be made.

The limitation of this approach is that slight variations in the assignment of constraints might result in similar model fit. The novel LMG approach to program evaluation is intentionally subjective; the pattern of equality constraints a researcher selects reflects her/his expectations. As such, scientific rigor must be brought to bear to test the veracity of these expectations. In other words, the researcher must conduct tests that can disprove that the expectations are empirically supported. We placed a limit on the degree of degradation to the fit of the overall unconstrained model resulting from the placement of equality constraints; the chi-square difference test must be no lower than *p* = .20. We also required that constrained means demonstrate statistical separation, and that the separation is robust to the removal of outliers. Final constraints were evaluated by the full study group to achieve consensus.

We used three stages to fit these models: (1) unconstrained, (2) constrained, and (3) 99 percentile winsorization. In the first step, constraints are imposed only on the measurement model, allowing means at each wave to be freely estimated for each group. In the second step, we selectively placed equality constraints on the means. Most importantly, we sought to preserve patterns supporting our hypothesis of beneficial program effects (i.e., SC means greater than corresponding DM means) in one or both cohorts and eliminate patterns counter to that hypothesis (DM means greater than corresponding SC means^[[15]](#footnote-16)^). In addition, we used the following guidelines to select constraints. We did not assume gender equivalence and allowed equality constraints to differ in either direction across gender when warranted. Similarly, we did not pose a priori expectations regarding the development of dating violence across time, allowing equality constraints on means at different waves to differ in either direction when warranted. In the third step, we truncated the most extreme covariate adjusted scores at the 99^th^ percentile and reran the constrained model to determine the robustness of the solution to outliers.

Obtaining Effect Size Estimates

Relative risk ratio (RRR) is traditionally used as an effect size estimate for dichotomous outcomes (e.g., a behavior occurred or did not occur). Our outcomes are a composite of the frequency of many behaviors. Expressing scores in POMS units, which has a meaningful 0 value (no behaviors were reported), allows us to interpret RRR as a ratio of two continuous scores. In the final models, parameters were used to calculate the RRR between DM and SC conditions, within group and wave. Because the calculations are conducted using model parameters, correct standard errors are obtained, making it possible to report the margin of error in the estimates of specific program effect.

In the context of the large amount of missing data, we recommend that the smaller program effects be interpreted with caution. In addition to student-level and item-level missingness common to school-based surveys, there was substantial structural missingness due to the cohort assessment pattern, to school entry/exit, and to directed skip. In the presence of high levels of missing data, correctness of the assumption of MAR/missing completely at random (MCAR) missing data mechanism which is demanded by MI becomes critical. Note that an assertion that any shifts in the moments of imputed variables is the correct and desirable consequence of MAR information cannot be tested. The plausibility and magnitude of any shifts can be examined with diagnostic plots but is ultimately a question of expert judgement. For these data, nearly all shifts corresponded to a negligible effect size. Most exceptions occurred at 11^th^ grade, where analysis precautions were being used. Most of the effects reported here were an order of magnitude larger than visualized MAR effects. Thus, smaller effects should be approached with caution as some are on the same order of magnitude as visualized MAR shifts. Figure 6 shows the minimum, average, and maximum RRR across all four outcomes over time point and groups.

References

1. Niolon PH, Taylor BG, Latzman NE, Vivolo-Kantor AM, Valle LA, Tharp AT. Lessons learned in evaluating a multisite, comprehensive teen dating violence prevention strategy: Design and challenges of the evaluation of dating matters: Strategies to promote healthy teen relationships. *Psychol Vio*. 2016;6(3):452-458. doi:10.1037/vio0000043

2. DeGue S, Niolon PH, Estefan LF, et al. Effects of Dating Matters® on sexual violence and sexual harassment outcomes among middle school youth: A cluster-randomized controlled trial. *Prev Sci*. 2021;22(2):175-185.

3. Estefan LF, Vivolo-Kantor AM, Niolon PH, et al. Effects of the dating matters® comprehensive prevention model on health-and delinquency-related risk behaviors in middle school youth: A cluster-randomized controlled trial. *Prev Sci.* 2021;22(2):163-174.

4. Niolon PH, Vivolo-Kantor AM, Tracy AJ, et al. An RCT of Dating Matters: Effects on Teen Dating Violence and Relationship Behaviors. *Am J Prev Med*. 2019;57(1):13-23. doi:10.1016/j.amepre.2019.02.022

5. Vivolo-Kantor AM, Niolon PH, Estefan LF, et al. Middle school effects of the Dating Matters® comprehensive teen dating violence prevention model on physical violence, bullying, and cyberbullying: A cluster-randomized controlled trial. *Prev Sci*. 2021;22(2):151-161.

6. Huque MH, Carlin JB, Simpson JA, Lee KJ. A comparison of multiple imputation methods for missing data in longitudinal studies. *BMC Med Res Methodol*. 2018;18(1):1-16.

7. Lee KJ, Carlin JB. Multiple imputation for missing data: fully conditional specification versus multivariate normal imputation. *Am J Epidemiol*. 2010;171(5):624-632.

8. Murray JS. Multiple imputation: a review of practical and theoretical findings. *Statistical Science*. 2018;33(2):142-159.

9. Collins LM, Schafer JL, Kam C-M. A comparison of inclusive and restrictive strategies in modern missing data procedures. *Psychol Methods*. 2001;6(4):330.

10. Howard WJ, Rhemtulla M, Little TD. Using Principal Components as Auxiliary Variables in Missing Data Estimation. *Multivariate Behav Res*. 2015;50(3):285-99. doi:10.1080/00273171.2014.999267

11. Lang KM, Little TD. Principled missing data treatments. *Prev Sci*. 2018;19(3):284-294.

12. Hippel PTv. How to Impute Interactions, Squares, and other Transformed Variables. *Sociol Methodol*. 2009;39(1):265-291. doi:doi:10.1111/j.1467-9531.2009.01215.x

13. Van Buuren S, Groothuis-Oudshoorn K. mice: Multivariate imputation by chained equations in R. *J Stat Soft*. 2011;45:1-67.

14. Van Buuren S. *Flexible imputation of missing data*. CRC press; 2018.

15. Nguyen CD, Carlin JB, Lee KJ. Model checking in multiple imputation: an overview and case study.

*Emerg Themes Epidemiol*. 2017;14(1):1-12.

16. What Works Clearinghouse standards handbook, version 4.1 (2020).

17. Yzerbyt VY, Muller D, Judd CM. Adjusting researchers’ approach to adjustment: On the use of covariates when testing interactions. *J Exp Soc Psychol*. 2004;40(3):424-431.

18. Little TD. *Longitudinal structural equation modeling*. Guilford Press; 2013.

19. Muthén L, Muthén B. Mplus user’s guide (Eighth edition). *Los Angeles, CA*. 1998-2017;

20. Little TD, Bontempo D, Rioux C, Tracy A. On the merits of longitudinal multiple group modelling: an alternative to multilevel modelling for intervention evaluations. *International Journal of Research & Method in Education*. 2021:1-13.

Appendix A. Supplementary Tables and Figures

eTable 1. Descriptive statistics for item average composites for outcome measures, prior to covariate adjustment.

|  |  | TDVP | | | | | | | | | | | |
| --- | --- | --- | --- | --- | --- | --- | --- | --- | --- | --- | --- | --- | --- |
|  | N | Grade 9 | Std. Dev | Minimum | Maximum | Grade 10 | Std. Dev | Minimum | Maximum | Grade 11 | Std. Dev | Minimum | Maximum |
| Female, Cohort 3, SC | 36500 | 1.22 | 0.23 | 1.00 | 2.45 | 1.28 | 0.26 | 1.00 | 2.74 | 1.29 | 0.22 | 1.00 | 2.94 |
| Female, Cohort 3, DM | 38300 | 1.20 | 0.22 | 1.00 | 3.03 | 1.28 | 0.28 | 1.00 | 2.94 | 1.27 | 0.20 | 1.00 | 2.29 |
| Female, Cohort 4, SC | 34400 | 1.19 | 0.18 | 1.00 | 3.00 | 1.22 | 0.24 | 1.00 | 2.74 | - | - | - | - |
| Female, Cohort 4, DM | 35300 | 1.22 | 0.22 | 1.00 | 2.81 | 1.23 | 0.25 | 1.00 | 2.87 | - | - | - | - |
| Male, Cohort 3, SC | 36900 | 1.18 | 0.26 | 1.00 | 3.03 | 1.25 | 0.26 | 1.00 | 3.03 | 1.22 | 0.18 | 1.00 | 2.45 |
| Male, Cohort 3, DM | 36300 | 1.16 | 0.22 | 1.00 | 2.97 | 1.27 | 0.27 | 1.00 | 3.06 | 1.23 | 0.18 | 1.00 | 2.29 |
| Male, Cohort 4, SC | 34700 | 1.16 | 0.18 | 1.00 | 2.45 | 1.20 | 0.20 | 1.00 | 2.48 | - | - | - | - |
| Male, Cohort 4, DM | 31600 | 1.16 | 0.22 | 1.00 | 2.77 | 1.21 | 0.25 | 1.00 | 2.77 | - | - | - | - |
| Total | 284000 | 1.19 | 0.22 | 1.00 | 3.03 | 1.24 | 0.25 | 1.00 | 3.06 | 1.26 | 0.19 | 1.00 | 2.94 |
|  |  | TDVV | | | | | | | | | | | |
|  | N | Grade 9 | Std. Dev | Minimum | Maximum | Grade 10 | Std. Dev | Minimum | Maximum | Grade 11 | Std. Dev | Minimum | Maximum |
| Female, Cohort 3, SC | 36500 | 1.23 | 0.24 | 1.00 | 3.13 | 1.27 | 0.24 | 1.00 | 2.87 | 1.33 | 0.24 | 1.00 | 2.94 |
| Female, Cohort 3, DM | 38300 | 1.22 | 0.25 | 1.00 | 3.35 | 1.28 | 0.26 | 1.00 | 2.97 | 1.31 | 0.22 | 1.00 | 2.48 |
| Female, Cohort 4, SC | 34400 | 1.19 | 0.19 | 1.00 | 3.23 | 1.21 | 0.21 | 1.00 | 2.58 | - | - | - | - |
| Female, Cohort 4, DM | 35300 | 1.23 | 0.24 | 1.00 | 3.19 | 1.23 | 0.23 | 1.00 | 2.74 | - | - | - | - |
| Male, Cohort 3, SC | 36900 | 1.23 | 0.29 | 1.00 | 3.23 | 1.27 | 0.27 | 1.00 | 3.16 | 1.30 | 0.22 | 1.00 | 2.55 |
| Male, Cohort 3, DM | 36300 | 1.22 | 0.27 | 1.00 | 3.19 | 1.30 | 0.29 | 1.00 | 3.10 | 1.30 | 0.21 | 1.00 | 2.35 |
| Male, Cohort 4, SC | 34700 | 1.19 | 0.19 | 1.00 | 2.77 | 1.23 | 0.21 | 1.00 | 2.52 | - | - | - | - |
| Male, Cohort 4, DM | 31600 | 1.20 | 0.28 | 1.00 | 3.52 | 1.24 | 0.26 | 1.00 | 3.10 | - | - | - | - |
| Total | 284000 | 1.21 | 0.25 | 1.00 | 3.52 | 1.25 | 0.25 | 1.00 | 3.16 | 1.31 | 0.20 | 1.00 | 2.94 |
|  |  | NCRS | | | | | | | | | | | |
|  | N | Grade 9 | Std. Dev | Minimum | Maximum | Grade 10 | Std. Dev | Minimum | Maximum | Grade 11 | Std. Dev | Minimum | Maximum |
| Female, Cohort 3, SC | 36500 | 1.84 | 0.59 | 1.00 | 5.00 | 1.83 | 0.58 | 1.00 | 4.33 | 1.84 | 0.50 | 1.00 | 4.00 |
| Female, Cohort 3, DM | 38300 | 1.79 | 0.53 | 1.00 | 4.67 | 1.84 | 0.54 | 1.00 | 3.92 | 1.83 | 0.50 | 1.00 | 4.08 |
| Female, Cohort 4, SC | 34400 | 1.77 | 0.52 | 1.00 | 3.75 | 1.73 | 0.57 | 1.00 | 5.00 | - | - | - | - |
| Female, Cohort 4, DM | 35300 | 1.85 | 0.52 | 1.00 | 4.00 | 1.75 | 0.54 | 1.00 | 4.17 | - | - | - | - |
| Male, Cohort 3, SC | 36900 | 1.75 | 0.52 | 1.00 | 4.08 | 1.72 | 0.50 | 1.00 | 4.00 | 1.69 | 0.43 | 1.00 | 4.08 |
| Male, Cohort 3, DM | 36300 | 1.68 | 0.49 | 1.00 | 4.08 | 1.75 | 0.53 | 1.00 | 3.83 | 1.74 | 0.48 | 1.00 | 3.67 |
| Male, Cohort 4, SC | 34700 | 1.65 | 0.46 | 1.00 | 3.58 | 1.65 | 0.50 | 1.00 | 4.42 | - | - | - | - |
| Male, Cohort 4, DM | 31600 | 1.70 | 0.49 | 1.00 | 4.17 | 1.63 | 0.46 | 1.00 | 3.92 | - | - | - | - |
| Total | 284000 | 1.76 | 0.52 | 1.00 | 5.00 | 1.74 | 0.53 | 1.00 | 5.00 | 1.75 | 0.44 | 1.00 | 4.08 |
|  |  | PRS | | | | | | | | | | | |
|  | N | Grade 9 | Std. Dev | Minimum | Maximum | Grade 10 | Std. Dev | Minimum | Maximum | Grade 11 | Std. Dev | Minimum | Maximum |
| Female, Cohort 3, SC | 36500 | 3.08 | 0.68 | 1.00 | 4.00 | 3.08 | 0.65 | 1.00 | 4.00 | 3.17 | 0.58 | 1.00 | 4.00 |
| Female, Cohort 3, DM | 38300 | 3.09 | 0.64 | 1.00 | 4.00 | 3.12 | 0.66 | 1.00 | 4.00 | 3.15 | 0.58 | 1.00 | 4.00 |
| Female, Cohort 4, SC | 34400 | 3.16 | 0.65 | 1.00 | 4.00 | 3.08 | 0.64 | 1.00 | 4.00 | - | - | - | - |
| Female, Cohort 4, DM | 35300 | 3.16 | 0.61 | 1.00 | 4.00 | 3.13 | 0.66 | 1.00 | 4.00 | - | - | - | - |
| Male, Cohort 3, SC | 36900 | 3.08 | 0.69 | 1.00 | 4.00 | 3.18 | 0.64 | 1.00 | 4.00 | 3.23 | 0.55 | 1.00 | 4.00 |
| Male, Cohort 3, DM | 36300 | 3.12 | 0.66 | 1.00 | 4.00 | 3.12 | 0.66 | 1.00 | 4.00 | 3.21 | 0.55 | 1.00 | 4.00 |
| Male, Cohort 4, SC | 34700 | 3.18 | 0.66 | 1.00 | 4.00 | 3.14 | 0.64 | 1.00 | 4.00 | - | - | - | - |
| Male, Cohort 4, DM | 31600 | 3.15 | 0.62 | 1.00 | 4.00 | 3.22 | 0.64 | 1.00 | 4.00 | - | - | - | - |
| Total | 284000 | 3.13 | 0.65 | 1.00 | 4.00 | 3.13 | 0.65 | 1.00 | 4.00 | 3.18 | 0.55 | 1.00 | 4.00 |

Note: The sample size for this table reflects the descriptive statistics in the student survey data, stacked by 100 imputations. TDVP = Teen Dating Violence Perpetration, TDVV = Teen Dating Violence Victimization, NCRS = Negative Conflict Resolution Strategies, PRS = Positive Relationship Skills.

eTable 2. Assignment of survey items to analysis “parcels” for latent variable modeling.

| **Construct (Parcel #)** | **Items in Parcel** |
| --- | --- |
| Teen Dating Violence Perpetration (TDVP1) | I did something to make him/her feel jealous. I brought up something bad he/she had done in the past. I said things just to make him/her angry. I spoke to him/her in a hostile or mean tone of voice. I insulted him/her with putdowns. I ridiculed or made fun of him/her in front of others. I kept track of who he/she was with and where he/she was. I blamed him/her for the problem. I accused him/her of flirting with another girl/guy. I threatened to end the relationship.  I threw something at him/her. I kicked, hit or punched him/her. I slapped him/her or pulled his/her hair. I pushed, shoved, or shook him/her. I destroyed or theatened to destroy something he/she valued. I deliberately tried to frighten him/her. I threatened to hurt him/her. I threatened to hit him/her or throw something at him/her. |
| Teen Dating Violence Perpetration (TDVP2) | I tried to turn his/her friends against him/her. I said things to his/her friends about him/her to turn them against him/her. I spread rumors about him/her. I touched him/her sexually when he/she didn't want me to. I forced him/her to have sex when he/she didn't want to. I threatened him/her in an attempt to have sex with him/her. I kissed him/her when he/she didn't want me to. |
| Teen Dating Violence Perpetration (TDVP3) | I threatened him/her with a knife or gun (including waving or pointing a knife). I scratched him/her and/or bent his/her fingers. I choked him/her. I used a knife or fired a gun. I burned him/her. I bit him/her. |
| Teen Dating Violence Victimization (TDVV1) | He/she did something to make me feel jealous. He/she brought up something bad I had done in the past. He/she said things just to make me angry. He/she spoke to me in a hostile or mean tone of voice. He/she insulted me with putdowns. He/she ridiculed or made fun of me in front of others. He/she kept track of who I was with and where I was. He/she blamed me for the problem. He/she accused me of flirting with another girl/guy. He/she threatened to end the relationship.  He/she threw something at me. He/she kicked, hit or punched me. He/she slapped me or pulled my hair. He/she pushed, shoved, or shook me. He/she destroyed or theatened to destroy something I valued. He/she deliberately tried to frighten me. He/she threatened to hurt me. He/she threatened to hit me or throw something at me. |
| Teen Dating Violence Victimization (TDVV2) | He/she tried to turn my friends against me. He/she said things to my friends about me to turn them against me. He/she spread rumors about me. He/she touched me sexually when I didn't want him/her to. He/she forced me to have sex when I didn't want to. He/she threatened me in an attempt to have sex with me. He/she kissed me when I didn't want him/her to. |
| Teen Dating Violence Victimization (TDVV3) | He/she threatened me with a knife or gun (including waving or pointing a knife). He/she scratched me and/or bent my fingers. He/she choked me. He/she used a knife or fired a gun. He/she burned me. He/she bit me. |
| Negative Conflict Resolution Strategies (NCRS1) | Remaining silent for long periods of time. Launching personal attacks. Getting carried away and saying things that aren't meant. Withdrawing, acting distant, and not interested. |
| Negative Conflict Resolution Strategies (NCRS2) | Not being willing to stick up for myself. Throwing insults and digs. Being too compliant. Exploding and getting out of control. |
| Negative Conflict Resolution Strategies (NCRS3) | Reaching a limit, shutting down, and refusing to talk any further. Tuning the other person out. Giving in with little attempt to present my side of the issue. Not defending my position. |
| Positive Relationship Skills (PRS1) | My boyfriend/girlfriend is/was honest and truthful with me. When I have a serious disagreement with my boyfriend/girlfriend, we discuss(ed) it respectfully. |
| Positive Relationship Skills (PRS2) | My boyfriend/girlfriend and I work(ed) as a team. |
| Positive Relationship Skills (PRS3) | My boyfriend/girlfriend and I are/were good at working out our differences. |

eTable 3. Equivalence evaluation across baseline outcome levels and selected demographic and other characteristics.

|  |  | Female, Cohort 3, SC | Female, Cohort 3, DM |  | Female, Cohort 4, SC | Female, Cohort 4, DM |  |
| --- | --- | --- | --- | --- | --- | --- | --- |
|  | N | 365 | 383 |  | 344 | 353 |  |
|  |  | Mean (SD)  or % | Mean (SD)  or % | Hedge's g or Cox's d | Mean (SD)  or % | Mean (SD)  or % | Hedge's g or Cox's d |
|  | Relative age difference | -0.08 (1.00) | -0.04 (0.97) | 0.04 | -0.08 (0.95) | -0.07 (0.90) | 0.00 |
|  | Hispanic (any race) | 30% | 30% | -0.02 | 33% | 29% | -0.13 |
|  | Black or African American | 49% | 50% | 0.03 | 49% | 50% | 0.02 |
|  | Asian | 7% | 5% | -0.24 | 8% | 7% | -0.08 |
|  | White | 2% | 5% | 0.63 | 3% | 6% | 0.48 |
|  | American Indian or Alaska native | 2% | 2% | -0.10 | 0% | 1% | n/a |
|  | Bi/multiracial | 10% | 9% | -0.10 | 7% | 8% | 0.06 |
| Baseline (Grade 6 Fall) | Teen Dating Violence Perpetration | 1.22 (0.29) | 1.22 (0.31) | 0.01 | 1.19 (0.26) | 1.21 (0.30) | 0.07 |
|  | Teen Dating Violence Victimization | 1.22 (0.28) | 1.22 (0.30) | 0.02 | 1.19 (0.25) | 1.20 (0.27) | 0.04 |
|  | Negative Conflict Resolution Strategies | 1.91 (0.59) | 1.94 (0.59) | 0.04 | 1.82 (0.55) | 1.89 (0.61) | 0.13 |
|  | Positive Relationship Skills | 2.73 (0.87) | 2.74 (0.89) | 0.01 | 2.62 (0.88) | 2.75 (0.89) | 0.15 |
| Grade 9 | Single mother/father | 27% | 29% | 0.06 | 26% | 28% | 0.06 |
|  | Two biological parents | 15% | 14% | -0.03 | 13% | 17% | 0.18 |
|  | Stepparent and biological parent | 13% | 13% | -0.01 | 15% | 12% | -0.12 |
|  | Foster mother and/or foster father | 0% | 1% | 0.21 | 1% | 0% | -0.62 |
|  | All others | 44% | 43% | -0.03 | 45% | 43% | -0.06 |
|  | In school survey mode | 38% | 36% | -0.05 | 33% | 29% | -0.11 |
|  | Witnessed violence | 42% | 41% | -0.02 | 38% | 42% | 0.10 |
|  | Relative survey administration lag | 7.93 (7.30) | 7.79 (7.14) | -0.02 | 3.21 (4.61) | 3.05 (4.62) | -0.03 |
| Grade 10 | Single mother/father | 32% | 30% | -0.05 | 32% | 30% | -0.06 |
|  | Two biological parents | 18% | 18% | 0.00 | 17% | 21% | 0.16 |
|  | Stepparent and biological parent | 11% | 11% | 0.04 | 13% | 12% | -0.06 |
|  | Foster mother and/or foster father | 0% | 1% | 0.40 | 0% | 0% | 0.25 |
|  | All others | 39% | 40% | 0.02 | 38% | 37% | -0.03 |
|  | In school survey mode | 26% | 29% | 0.09 | 22% | 22% | 0.00 |
|  | Witnessed violence | 43% | 40% | -0.07 | 29% | 32% | 0.09 |
|  | Relative survey administration lag | 2.12 (4.24) | 2.00 (3.52) | -0.03 | -11.75 (6.29) | -13.00 (5.75) | -0.17 |
| Grade 11 | Single mother/father | 27% | 30% | 0.08 | - | - | - |
|  | Two biological parents | 24% | 19% | -0.17 | - | - | - |
|  | Stepparent and biological parent | 11% | 11% | 0.01 | - | - | - |
|  | Foster mother and/or foster father | 1% | 1% | 0.15 | - | - | - |
|  | All others | 38% | 40% | 0.04 | - | - | - |
|  | In school survey mode | 16% | 19% | 0.13 | - | - | - |
|  | Witnessed violence | 33% | 34% | 0.03 | - | - | - |
|  | Relative survey administration lag | -12.33 (6.39) | -13.10 (5.66) | -0.11 | - | - | - |
|  |  | Male, Cohort 3, SC | Male, Cohort 3, DM |  | Male, Cohort 4, SC | Male, Cohort 4, DM |  |
|  | N | 369 | 363 |  | 347 | 316 |  |
|  |  | Mean (SD)  or % | Mean (SD)  or % | Hedge's g or Cox's d | Mean (SD)  or % | Mean (SD)  or % | Hedge's g or Cox's d |
|  | Relative age difference | 0.08 (0.96) | 0.08 (1.04) | 0.00 | -0.03 (0.97) | 0.15 (1.17) | **0.14** |
|  | Hispanic (any race) | 38% | 30% | **-0.22** | 31% | 28% | **-0.11** |
|  | Black or African American | 49% | 55% | **0.15** | 48% | 48% | -0.01 |
|  | Asian | 6% | 4% | ***-0.26*** | 11% | 9% | **-0.11** |
|  | White | 2% | 5% | ***0.66*** | 5% | 5% | **0.06** |
|  | American Indian or Alaska native | 1% | 1% | **-0.17** | 2% | 1% | ***-0.62*** |
|  | Bi/multiracial | 4% | 5% | **0.14** | 3% | 10% | ***0.68*** |
| Baseline (Grade 6 Fall) | Teen Dating Violence Perpetration | 1.20 (0.30) | 1.20 (0.29) | 0.01 | 1.17 (0.26) | 1.17 (0.28) | -0.01 |
|  | Teen Dating Violence Victimization | 1.23 (0.30) | 1.22 (0.28) | -0.01 | 1.20 (0.28) | 1.20 (0.28) | 0.00 |
|  | Negative Conflict Resolution Strategies | 1.81 (0.60) | 1.83 (0.57) | 0.04 | 1.71 (0.54) | 1.75 (0.55) | **0.07** |
|  | Positive Relationship Skills | 2.80 (0.89) | 2.91 (0.85) | **0.13** | 2.88 (0.89) | 2.87 (0.90) | -0.01 |
| Grade 9 | Single mother/father | 26% | 28% | 0.04 | 27% | 27% | -0.02 |
|  | Two biological parents | 20% | 22% | **0.09** | 20% | 22% | **0.08** |
|  | Stepparent and biological parent | 11% | 11% | -0.01 | 11% | 11% | -0.03 |
|  | Foster mother and/or foster father | 0% | 1% | **0.08** | 0% | 0% | 0.05 |
|  | All others | 42% | 39% | **-0.09** | 41% | 40% | -0.03 |
|  | In school survey mode | 39% | 32% | **-0.19** | 32% | 31% | -0.03 |
|  | Witnessed violence | 48% | 44% | **-0.10** | 41% | 38% | **-0.08** |
|  | Relative survey administration lag | 7.27 (6.63) | 8.23 (6.44) | **0.12** | 3.14 (4.57) | 2.68 (4.01) | **-0.09** |
| Grade 10 | Single mother/father | 31% | 30% | -0.03 | 29% | 27% | -0.05 |
|  | Two biological parents | 23% | 24% | 0.05 | 22% | 21% | -0.02 |
|  | Stepparent and biological parent | 9% | 8% | -0.02 | 7% | 7% | -0.03 |
|  | Foster mother and/or foster father | 0% | 1% | ***0.42*** | 0% | 0% | 0.02 |
|  | All others | 37% | 37% | -0.01 | 42% | 45% | **0.07** |
|  | In school survey mode | 29% | 27% | **-0.06** | 25% | 25% | 0.00 |
|  | Witnessed violence | 43% | 42% | -0.02 | 35% | 32% | **-0.08** |
|  | Relative survey administration lag | 2.06 (4.05) | 2.04 (3.73) | 0.00 | -11.40 (6.10) | -12.56 (5.00) | **-0.17** |
| Grade 11 | Single mother/father | 26% | 27% | 0.03 | - | - | - |
|  | Two biological parents | 28% | 30% | 0.05 | - | - | - |
|  | Stepparent and biological parent | 9% | 8% | -0.02 | - | - | - |
|  | Foster mother and/or foster father | 1% | 1% | 0.01 | - | - | - |
|  | All others | 37% | 35% | **-0.06** | - | - | - |
|  | In school survey mode | 19% | 19% | 0.00 | - | - | - |
|  | Witnessed violence | 30% | 33% | **0.08** | - | - | - |
|  | Relative survey administration lag | -12.57 (5.34) | -12.76 (5.98) | -0.03 | - | - | - |

Note: Nonequivalence evaluation of baseline levels of outcomes are drawn from the 100 imputed datasets. Other characteristics included as covariates draw from grand mean composites of the imputed data. Cohort 4 was in Grade 10 and Cohort 3 was in Grade 11 during the final year of the study, resulting in early survey administration dates for that wave. According to the recommendations of the What Works Clearinghous,^16^ the absolute value of Cox’s *d* (used with categorical variables) or Hedge’s *g* (used with continuous variables) higher than 0.05 but less than 0.25 (bolded) represent nonequivalence that is correctable through covariate adjustment. Hedge’s *g* or Cox’s *d* values greater than or equal to 0.25 (bolded and italicized) indicate characteristics that may pose a risk of unintended bias and must be considered when interpreting the results. However, it is useful to note that the comparison of proportions near the lower bound (0%) can produce a very large test statistic but represent very little practical difference across treatment conditions.

eTable 4. Model Results: Teen Dating Violence Perpetration

| Unconstrained | | | | Constrained | | | | Difference | | |
| --- | --- | --- | --- | --- | --- | --- | --- | --- | --- | --- |
| Chi-square | df | RMSEA | SRMR | Chi-square | df | RMSEA | SRMR | Chi-square | df | *p*-value |
| 438.47 | 288 | 0.04 | 0.07 | 452.33 | 304 | 0.04 | 0.07 | 13.87 | 16 | 0.609 |

| Constrained Means | | |  | Pairwise Diff Tests (adjacent) | | |
| --- | --- | --- | --- | --- | --- | --- |
| Rank | Mean | # in Band |  |  | *Wald* | *p*-value |
| 1 | 1.21 | 5 |  | 1 v 2 | -0.59 | 0.000 |
| 2 | 1.80 | 8 |  | 2 v 3 | -1.11 | 0.000 |
| 3 | 2.92 | 5 |  | 3 v 4 | -1.53 | 0.000 |
| 4 | 4.44 | 2 |  |  |  |  |

*Model-Estimated Means*

|  | N | Grade 9 | Grade 10 | Grade 11 |
| --- | --- | --- | --- | --- |
| Unconstrained |  |  |  |  |
| SC Female - Cohort 3 | 365 | 1.78 | 2.16 | 4.64 |
| DM Female - Cohort 3 | 383 | 1.71 | 1.49 | 4.41 |
| SC Female - Cohort 4 | 344 | 1.78 | 1.90 |  |
| DM Female - Cohort 4 | 353 | 1.88 | 1.07 |  |
| SC Male - Cohort 3 | 369 | 1.80 | 2.82 | 2.85 |
| DM Male - Cohort 3 | 363 | 1.22 | 3.42 | 2.93 |
| SC Male - Cohort 4 | 347 | 1.70 | 2.85 |  |
| DM Male - Cohort 4 | 316 | 1.25 | 1.42 |  |
| Constrained |  |  |  |  |
| SC Female - Cohort 3 | 365 | 1.80 | 1.80 | 4.44 |
| DM Female - Cohort 3 | 383 | 1.80 | 1.21 | 4.44 |
| SC Female - Cohort 4 | 344 | 1.80 | 1.80 |  |
| DM Female - Cohort 4 | 353 | 1.80 | 1.21 |  |
| SC Male - Cohort 3 | 369 | 1.80 | 2.92 | 2.92 |
| DM Male - Cohort 3 | 363 | 1.21 | 2.92 | 2.92 |
| SC Male - Cohort 4 | 347 | 1.80 | 2.92 |  |
| DM Male - Cohort 4 | 316 | 1.21 | 1.21 |  |

*Program Effect Size Estimates*

|  | Grade 9 | Grade 10 | Grade 11 |
| --- | --- | --- | --- |
| Female - Cohort 3 |  |  |  |
| RRR | 0.00 | 32.78 | 0.00 |
| SE | n/a | 8.79 | n/a |
| Lower limit (95% CI) | 0.00 | 15.55 | 0.00 |
| Upper limit (95% CI) | 0.00 | 50.01 | 0.00 |
| Female - Cohort 4 |  |  |  |
| RRR | 0.00 | 32.78 |  |
| SE | n/a | 8.79 |  |
| Lower limit (95% CI) | 0.00 | 15.55 |  |
| Upper limit (95% CI) | 0.00 | 50.01 |  |
| Male - Cohort 3 |  |  |  |
| RRR | 32.78 | 0.00 | 0.00 |
| SE | 8.79 | n/a | n/a |
| Lower limit (95% CI) | 15.55 | 0.00 | 0.00 |
| Upper limit (95% CI) | 50.01 | 0.00 | 0.00 |
| Male - Cohort 4 |  |  |  |
| RRR | 32.78 | 58.56 |  |
| SE | 8.79 | 5.49 |  |
| Lower limit (95% CI) | 15.55 | 47.80 |  |
| Upper limit (95% CI) | 50.01 | 69.32 |  |

Note: SC = Standard of Care, DM = *Dating Matters*^®^, RRR = relative risk ratio, CI = confidence interval

eTable 5. Model Results: Teen Dating Violence Victimization

| Unconstrained | | | | Constrained | | | | Difference | | |
| --- | --- | --- | --- | --- | --- | --- | --- | --- | --- | --- |
| Chi-square | df | RMSEA | SRMR | Chi-square | df | RMSEA | SRMR | Chi-square | df | *p*-value |
| 277.29 | 288 | 0.00 | 0.05 | 296.47 | 304 | 0.00 | 0.05 | 19.18 | 16 | 0.259 |

| Constrained Means | | |  | Pairwise Diff Tests (adjacent) | | |
| --- | --- | --- | --- | --- | --- | --- |
| Rank | Mean | # in Band |  |  | *Wald* | *p*-value |
| 1 | 1.34 | 4 |  | 1 v 2 | -0.64 | 0.005 |
| 2 | 1.98 | 6 |  | 2 v 3 | -1.04 | 0.000 |
| 3 | 3.02 | 6 |  | 3 v 4 | -1.54 | 0.000 |
| 4 | 4.55 | 4 |  |  |  |  |

*Model-Estimated Means*

|  | N | Grade 9 | Grade 10 | Grade 11 |
| --- | --- | --- | --- | --- |
| Unconstrained |  |  |  |  |
| SC Female - Cohort 3 | 365 | 2.20 | 3.18 | 4.76 |
| DM Female - Cohort 3 | 383 | 2.27 | 2.23 | 5.32 |
| SC Female - Cohort 4 | 344 | 2.47 | 2.50 |  |
| DM Female - Cohort 4 | 353 | 2.20 | 1.52 |  |
| SC Male - Cohort 3 | 369 | 1.70 | 3.17 | 4.15 |
| DM Male - Cohort 3 | 363 | 1.16 | 3.53 | 4.19 |
| SC Male - Cohort 4 | 347 | 1.91 | 3.18 |  |
| DM Male - Cohort 4 | 316 | 1.11 | 1.55 |  |
| Constrained |  |  |  |  |
| SC Female - Cohort 3 | 365 | 1.98 | 3.02 | 4.55 |
| DM Female - Cohort 3 | 383 | 1.98 | 1.98 | 4.55 |
| SC Female - Cohort 4 | 344 | 3.02 | 3.02 |  |
| DM Female - Cohort 4 | 353 | 1.98 | 1.34 |  |
| SC Male - Cohort 3 | 369 | 1.98 | 3.02 | 4.55 |
| DM Male - Cohort 3 | 363 | 1.34 | 3.02 | 4.55 |
| SC Male - Cohort 4 | 347 | 1.98 | 3.02 |  |
| DM Male - Cohort 4 | 316 | 1.34 | 1.34 |  |

*Program Effect Size Estimates*

|  | Grade 9 | Grade 10 | Grade 11 |
| --- | --- | --- | --- |
| Female - Cohort 3 |  |  |  |
| RRR | 0.00 | 34.44 | 0.00 |
| SE | n/a | 5.25 | n/a |
| Lower limit (95% CI) | 0.00 | 24.15 | 0.00 |
| Upper limit (95% CI) | 0.00 | 44.73 | 0.00 |
| Female - Cohort 4 |  |  |  |
| RRR | 34.44 | 55.63 |  |
| SE | 5.25 | 6.61 |  |
| Lower limit (95% CI) | 24.15 | 42.67 |  |
| Upper limit (95% CI) | 44.73 | 68.58 |  |
| Male - Cohort 3 |  |  |  |
| RRR | 32.32 | 0.00 | 0.00 |
| SE | 10.43 | n/a | n/a |
| Lower limit (95% CI) | 11.88 | 0.00 | 0.00 |
| Upper limit (95% CI) | 52.77 | 0.00 | 0.00 |
| Male - Cohort 4 |  |  |  |
| RRR | 32.32 | 55.63 |  |
| SE | 10.43 | 6.61 |  |
| Lower limit (95% CI) | 11.88 | 42.67 |  |
| Upper limit (95% CI) | 52.77 | 68.58 |  |

Note: SC = Standard of Care, DM = *Dating Matters*^®^, RRR = relative risk ratio, CI = confidence interval

eTable 6. Model Results: Negative Conflict Resolution Strategies

| Unconstrained | | | | Constrained | | | | Difference | | |
| --- | --- | --- | --- | --- | --- | --- | --- | --- | --- | --- |
| Chi-square | df | RMSEA | SRMR | Chi-square | df | RMSEA | SRMR | Chi-square | df | *p*-value |
| 64.33 | 288 | 0.00 | 0.02 | 85.75 | 305 | 0.00 | 0.03 | 21.42 | 17 | 0.208 |

| Constrained Means | | |  | Pairwise Diff Tests (adjacent) | | |
| --- | --- | --- | --- | --- | --- | --- |
| Rank | Mean | # in Band |  |  | *Wald* | *p*-value |
| 1 | 11.40 | 3 |  | 1 v 2 | -1.59 | 0.003 |
| 2 | 12.99 | 6 |  | 2 v 3 | -1.68 | 0.000 |
| 3 | 14.67 | 11 |  |  |  |  |

*Model-Estimated Means*

|  | N | Grade 9 | Grade 10 | Grade 11 |
| --- | --- | --- | --- | --- |
| Unconstrained |  |  |  |  |
| SC Female - Cohort 3 | 365 | 16.13 | 13.91 | 13.39 |
| DM Female - Cohort 3 | 383 | 13.25 | 15.15 | 13.05 |
| SC Female - Cohort 4 | 344 | 13.50 | 15.13 |  |
| DM Female - Cohort 4 | 353 | 16.97 | 12.81 |  |
| SC Male - Cohort 3 | 369 | 15.16 | 13.54 | 11.84 |
| DM Male - Cohort 3 | 363 | 11.58 | 14.86 | 13.33 |
| SC Male - Cohort 4 | 347 | 13.71 | 13.28 |  |
| DM Male - Cohort 4 | 316 | 14.12 | 10.47 |  |
| Constrained |  |  |  |  |
| SC Female - Cohort 3 | 365 | 14.67 | 14.67 | 12.99 |
| DM Female - Cohort 3 | 383 | 12.99 | 14.67 | 12.99 |
| SC Female - Cohort 4 | 344 | 14.67 | 14.67 |  |
| DM Female - Cohort 4 | 353 | 14.67 | 11.40 |  |
| SC Male - Cohort 3 | 369 | 14.67 | 14.67 | 12.99 |
| DM Male - Cohort 3 | 363 | 11.40 | 14.67 | 12.99 |
| SC Male - Cohort 4 | 347 | 14.67 | 12.99 |  |
| DM Male - Cohort 4 | 316 | 14.67 | 11.40 |  |

*Program Effect Size Estimates*

|  | Grade 9 | Grade 10 | Grade 11 |
| --- | --- | --- | --- |
| Female - Cohort 3 |  |  |  |
| RRR | 11.45 | 0.00 | 0.00 |
| SE | 2.62 | n/a | n/a |
| Lower limit (95% CI) | 6.32 | 0.00 | 0.00 |
| Upper limit (95% CI) | 16.59 | 0.00 | 0.00 |
| Female - Cohort 4 |  |  |  |
| RRR | 0.00 | 22.29 |  |
| SE | n/a | 3.11 |  |
| Lower limit (95% CI) | 0.00 | 16.19 |  |
| Upper limit (95% CI) | 0.00 | 28.39 |  |
| Male - Cohort 3 |  |  |  |
| RRR | 22.29 | 0.00 | 0.00 |
| SE | 3.11 | n/a | n/a |
| Lower limit (95% CI) | 16.19 | 0.00 | 0.00 |
| Upper limit (95% CI) | 28.39 | 0.00 | 0.00 |
| Male - Cohort 4 |  |  |  |
| RRR | 0.00 | 12.24 |  |
| SE | n/a | 3.93 |  |
| Lower limit (95% CI) | 0.00 | 4.54 |  |
| Upper limit (95% CI) | 0.00 | 19.94 |  |

Note: SC = Standard of Care, DM = *Dating Matters*^®^, RRR = relative risk ratio, CI = confidence interval

eTable 7. Model Results: Positive Relationship Skills

| Unconstrained | | | | Constrained | | | | Difference | | |
| --- | --- | --- | --- | --- | --- | --- | --- | --- | --- | --- |
| Chi-square | df | RMSEA | SRMR | Chi-square | df | RMSEA | SRMR | Chi-square | df | *p*-value |
| 44.79 | 288 | 0.00 | 0.02 | 64.98 | 304 | 0.00 | 0.03 | 20.19 | 16 | 0.212 |

| Constrained Means | | |  | Pairwise Diff Tests (adjacent) | | |
| --- | --- | --- | --- | --- | --- | --- |
| Rank | Mean | # in Band |  |  | *Wald* | *p*-value |
| 1 | 70.27 | 4 |  | 1 v 2 | -3.75 | 0.000 |
| 2 | 74.03 | 11 |  | 2 v 3 | -2.43 | 0.001 |
| 3 | 76.46 | 4 |  | 3 v 4 | -3.85 | 0.009 |
| 4 | 80.31 | 1 |  |  |  |  |

*Model-Estimated Means*

|  | N | Grade 9 | Grade 10 | Grade 11 |
| --- | --- | --- | --- | --- |
| Unconstrained |  |  |  |  |
| SC Female - Cohort 3 | 365 | 69.99 | 75.19 | 72.92 |
| DM Female - Cohort 3 | 383 | 68.43 | 78.15 | 73.37 |
| SC Female - Cohort 4 | 344 | 74.22 | 70.00 |  |
| DM Female - Cohort 4 | 353 | 72.09 | 75.63 |  |
| SC Male - Cohort 3 | 369 | 77.13 | 76.01 | 76.09 |
| DM Male - Cohort 3 | 363 | 72.98 | 75.57 | 74.15 |
| SC Male - Cohort 4 | 347 | 73.82 | 71.15 |  |
| DM Male - Cohort 4 | 316 | 73.47 | 80.27 |  |
| Constrained |  |  |  |  |
| SC Female - Cohort 3 | 365 | 70.27 | 74.03 | 70.27 |
| DM Female - Cohort 3 | 383 | 70.27 | 76.46 | 74.03 |
| SC Female - Cohort 4 | 344 | 74.03 | 70.27 |  |
| DM Female - Cohort 4 | 353 | 74.03 | 76.46 |  |
| SC Male - Cohort 3 | 369 | 74.03 | 76.46 | 74.03 |
| DM Male - Cohort 3 | 363 | 74.03 | 76.46 | 74.03 |
| SC Male - Cohort 4 | 347 | 74.03 | 74.03 |  |
| DM Male - Cohort 4 | 316 | 74.03 | 80.31 |  |

*Program Effect Size Estimates*

|  | Grade 9 | Grade 10 | Grade 11 |
| --- | --- | --- | --- |
| Female - Cohort 3 |  |  |  |
| RRR | 0.00 | 9.36 | 12.65 |
| SE | n/a | 2.88 | 2.24 |
| Lower limit (95% CI) | 0.00 | 3.71 | 8.26 |
| Upper limit (95% CI) | 0.00 | 15.00 | 17.04 |
| Female - Cohort 4 |  |  |  |
| RRR | 0.00 | 20.82 |  |
| SE | n/a | 2.83 |  |
| Lower limit (95% CI) | 0.00 | 15.27 |  |
| Upper limit (95% CI) | 0.00 | 26.37 |  |
| Male - Cohort 3 |  |  |  |
| RRR | 0.00 | 0.00 | 0.00 |
| SE | n/a | n/a | n/a |
| Lower limit (95% CI) | 0.00 | 0.00 | 0.00 |
| Upper limit (95% CI) | 0.00 | 0.00 | 0.00 |
| Male - Cohort 4 |  |  |  |
| RRR | 0.00 | 24.18 |  |
| SE | n/a | 5.40 |  |
| Lower limit (95% CI) | 0.00 | 13.60 |  |
| Upper limit (95% CI) | 0.00 | 34.77 |  |

Note: SC = standard-of-care, DM = *Dating Matters*^®^, RRR = relative risk ratio, CI = confidence interval. A higher score indicates a better outcome for this measure, but the RRR are scaled to parallel the other outcomes; a positive RRR reflects a desirable program effect, using a score of 100 as the reference value equivalent to zero risk.

1. Graham (2012) calls these targeted imputations the “impute once, analyze once” strategy, in contrast to “impute once, analyze many.” He argues for greater use of targeted imputation because the increasing degree of automation and high-level scripting tools that most commercial statistical packages provide for multiple imputation make this an increasingly feasible proposition. However, his concern focuses on issues of multicollinearity and estimation stability, issues that are addressed by the *PcAux* methodology used with these data. [↑](#footnote-ref-2)
2. To mitigate computational demands, the *mice* package offers a function called *quickPred* which excludes variables from each model’s predictor set if the pairwise association with the model’s outcome falls below a user-specified threshold. *PcAux* offers *pcQuickPred* which similarly permits exclusion of component scores. [↑](#footnote-ref-3)
3. All calculations, including any necessary dummy-coding and subsequent orthogonalization of interaction terms with main effects is automated. [↑](#footnote-ref-4)
4. We constructed time-invariant student demographic information (gender, birth date, race/Hispanic ethnicity) to stabilize the sample across the middle school evaluation, which utilized interim imputations conducted on only the middle school data when it became available, and the high school evaluations, which utilized imputations conducted on the entire set of middle and high school data. When there were discrepancies in the reported birth date, gender, and race/ethnicity responses over middle school waves, we used the most recently reported response. Missing data in these composite demographics were imputed after all middle school data had been collected, and final categories were assigned based on the preponderance of the imputed middle school values. These characteristics were subsequently treated as complete; missing data for these variables were not re-imputed after receiving high school data. We acknowledge that this reifies imputed placeholder values, which should not be thought of as “truth.” Most imputed race or gender values, however, had very clear patterns with a very large modal value so we have high confidence in the resulting composite as a robust grouping variable. [↑](#footnote-ref-5)
5. For the general imputation of these data, a customized branch of *PcAux* v0.0.9013 was used. The main customization provided for threshold testing principal component predictors for each model to exclude many low-value predictors from each FCS model in the chain. This feature has now been added to *PcAux* v0.0.9015. [↑](#footnote-ref-6)
6. Pmm has been observed to perform well with bounded and discrete data. While the use of pmm for ordinal and binary data has not been formally studied, we conducted a preliminary empirical study with simulated ordinal and binary items resembling our data. The results of this study supported the use of pmm for the DM dataset. With limited information due to very high levels of missing data, high skew ordinal variables (most participants endorsed no/never) and binary variables had the most plausible MAR shifts with pmm, as compared to ordinal and logistic regression. [↑](#footnote-ref-7)
7. In prior work with only the six middle school waves, middle-school IDs were used as moderators instead of site. [↑](#footnote-ref-8)
8. This mutually exclusive time-invariant categorization of race draws from students’ responses to the ethnicity survey item and the set of race survey items, supplemented by the student’s fill-in response, when available. Within each time point, endorsements of multiple race categories were coded as biracial. Any endorsement of Hispanic ethnicity, regardless of other race categories endorsed, resulted in a code of “Hispanic (any race).” When responses over time were not consistent, the most recently reported responses were used. Low-prevalence categories created problems with convergence in the covariate adjustment models. Therefore, Native Hawaiian/Other Pacific Islander, Native American/Alaska Native, White, and Other (not specified) were combined, leaving an indicator for non-Hispanic black race and a compilation of all other non-Hispanic racial categories, with Hispanic ethnicity (any race) as the comparison category. [↑](#footnote-ref-9)
9. We constructed time-varying guardianship status drawing from a set of “check all that apply” options to the item prompt: “Think about the grown-ups that live in your house. Mark circles to indicate all the grown-ups who live in your house.” Response options were as follows: mother, father, stepmother, stepfather, foster mother, foster father, grandmother, grandfather, aunt, uncle, and other. These responses were used to create two dichotomous indicators representing guardian status where two biological parents were contrasted with single parents, foster/stepparents (combined for covariate adjustment), and all others. [↑](#footnote-ref-10)
10. Students matriculated to many high schools and the dataset did not contain indicators of high school membership. In the high school evaluations, we chose not to adjust for middle school of origin. Rather, we adjusted for site to account for shared environmental context. [↑](#footnote-ref-11)
11. One dimension of the mode of high school survey administration was selected as relevant for covariate adjustment: the physical context (1= student took the survey in school or 0=otherwise). [↑](#footnote-ref-12)
12. Four dichotomous items were combined into a single dichotomous indicator, coded “1” if any witnessing of parental or community violence was reported. [↑](#footnote-ref-13)
13. Relative age was calculated as the number of years between the student’s month and day of birth and November 22 of the student’s Grade 6 academic year. Survey dates were converted to survey lag, measured in weeks, relative to May 28 of the relevant assessment year. [↑](#footnote-ref-14)
14. For a small number of models there was insufficient variability after trimming to permit estimation. When the trimmed model failed, the initial untrimmed model was used to produce adjusted values. [↑](#footnote-ref-15)
15. Like other principled discovery approaches, such as cluster analysis, latent growth modeling, latent class analysis, or latent transition analysis, LMG can produce multiple statistical solutions that describe the data well. Using constraint selection to eliminate counterintuitive effects (DM with poorer estimated outcomes than SC) limits our ability to identify unintended consequences of program participation. One strength of the LMG approach, however, is that assumptions underlying the selection of constraints can be empirically evaluated by examining model misfit. In other words, our test of global model fit is designed to safeguard against eliminating true counterintuitive program effects. [↑](#footnote-ref-16)
